# Computer-aided design of multi-target ligands at A_1_R, A_2A_R and PDE10A, key proteins in neurodegenerative diseases

**DOI:** 10.1186/s13321-017-0249-4

**Published:** 2017-12-30

**Authors:** Leen Kalash, Cristina Val, Jhonny Azuaje, María I. Loza, Fredrik Svensson, Azedine Zoufir, Lewis Mervin, Graham Ladds, José Brea, Robert Glen, Eddy Sotelo, Andreas Bender

**Affiliations:** 1 Department of Chemistry, Centre for Molecular Informatics, University of Cambridge, Lensfield Road, Cambridge, CB21EW UK; 2 Center for Research in Biological Chemistry and Molecular Materials (CIQUS), University of Santiago de Compostela, 15782 Santiago de Compostela, Spain; 3 Center for Research in Molecular Medicine and Chronic Diseases (CIMUS), University of Santiago de Compostela, 15782 Santiago de Compostela, Spain; 4 IOTA Pharmaceuticals Ltd, St Johns Innovation Centre, Cowley Road, Cambridge, CB40WS UK; 5 Discovery Sciences, AstraZeneca R&D, Cambridge Science Park, Cambridge, UK; 6 Department of Pharmacology, University of Cambridge, Tennis Court Road, Cambridge, CB21QJ UK; 7 Division of Computational and Systems Medicine, Department of Surgery and Cancer, Imperial College London, London, UK

**Keywords:** Multi-target ligands, Adenosine receptor ligands, PDE10A inhibitors, Target prediction, Drug design, Docking, QSAR

## Abstract

Compounds designed to display polypharmacology may have utility in treating complex diseases, where activity at multiple targets is required to produce a clinical effect. In particular, suitable compounds may be useful in treating neurodegenerative diseases by promoting neuronal survival in a synergistic manner via their *multi*-*target* activity at the adenosine A_1_ and A_2A_ receptors (A_1_R and A_2A_R) and phosphodiesterase 10A (PDE10A), which modulate intracellular cAMP levels. Hence, in this work we describe a computational method for the design of synthetically feasible ligands that bind to A_1_ and A_2A_ receptors and inhibit phosphodiesterase 10A (PDE10A), involving a retrosynthetic approach employing in silico target prediction and docking, which may be generally applicable to multi-target compound design at several target classes. This approach has identified 2-aminopyridine-3-carbonitriles as the first multi-target ligands at A_1_R, A_2A_R and PDE10A, by showing agreement between the ligand and structure based predictions at these targets. The series were synthesized via an efficient one-pot scheme and validated pharmacologically as A_1_R/A_2A_R–PDE10A ligands, with IC_50_ values of 2.4–10.0 μM at PDE10A and K_i_ values of 34–294 nM at A_1_R and/or A_2A_R. Furthermore, selectivity profiling of the synthesized 2-amino-pyridin-3-carbonitriles against other subtypes of both protein families showed that the multi-target ligand **8** exhibited a minimum of twofold selectivity over all tested off-targets. In addition, both compounds **8** and **16** exhibited the desired multi-target profile, which could be considered for further functional efficacy assessment, analog modification for the improvement of selectivity towards A_1_R, A_2A_R and PDE10A collectively, and evaluation of their potential synergy in modulating cAMP levels.

**Electronic supplementary material:**

The online version of this article (10.1186/s13321-017-0249-4) contains supplementary material, which is available to authorized users.

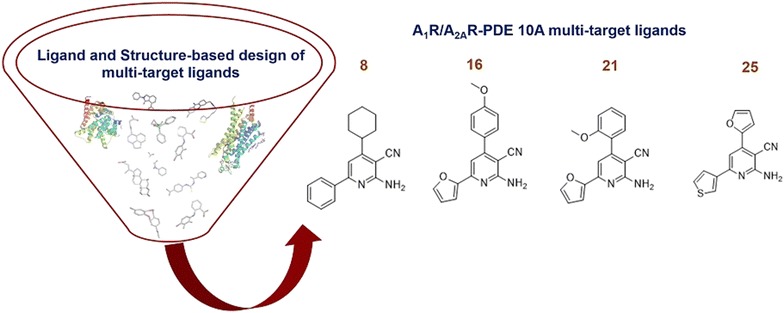

## Background

Neurodegeneration involves the progressive loss of the structure and function of neurons, which is common in Parkinson’s, Huntington’s disease and schizophrenia [1]. Recently, there has been substantial interest in the search for alternative non-dopamine (non-DA) based approaches for the treatment of neurodegenerative diseases, as the classical DA-based approaches have long been associated with many undesirable side effects such as dyskinesia, hallucinations, and on/off effects [2]. Given that the adenosine neuromodulation system (via the adenosine A_1_ and A_2A_ receptors) has been identified as a key target for the management of neurodegenerative diseases, this qualifies its targeting as a potential promising non-DA based treatment approach [3, 4]. Indeed, modulation of cAMP levels has proven to have benefits in neuronal survival in an adenosine receptor-dependent manner [5]. In addition, recent findings suggest that phosphodiesterase 10A (PDE10A) also plays a role in neurodegenerative diseases such as Parkinson’s, Huntington’s disease, and schizophrenia [6–8]. Inhibition of PDE10A resulting in maintenance of elevated intracellular cAMP concentrations, has been suggested to be effective in the treatment of these diseases. Thus multi-target ligands that bind to different adenosine receptors subtypes (A_1_ and A_2A_ receptors) while simultaneously inhibit PDE10A might be synergistic in modulating cAMP levels, which is of therapeutic potential for neurodegenerative diseases [9–11].

Conceptually, multi-target drugs work by creating a combination effect on multiple targets in the biological network simultaneously, which may (through e.g. synergistic effects) decrease the therapeutic dose required, thus increasing therapeutic efficacy, preventing drug resistance, and reducing target-related adverse effects [12–14]. Also, another advantage of multi-target drugs over other types of treatments such as combination therapies, is a reduced likelihood of drug–drug interactions [15, 16].

However, it remains a challenging task for medicinal chemists to design drugs with a specific multi-target profile and to achieve selectivity for specific targets over off-target effects with suitable pharmacokinetic properties [17, 18]. In fact, the field of multi-target drug design has recently become an active field of research in the pharmaceutical industry, where around 20 designed multi-target drugs have either reached advanced development stages or are already approved [14, 19, 20].

In particular, for Central Nervous System (CNS) diseases, there has been growing interest in exploiting the multi-target profiles of existing compounds to investigate their potential applicability as drugs. For example, multi-target profiles of drugs and drug candidates affecting the dopaminergic system have been investigated. Examples include Aripiprazole, Amitriptyline, Chlorpromazine, and Clozapine [21]. In addition, various multi-target based virtual screening protocols for multi-target drug design have been developed [13, 22–24]. Examples of ligand-based protocols include in silico target prediction and Chemogenomic and pharmacophore-based approaches, which resulted in the discovery of CNS drugs with multi-target combinations such as MAO-A/MAO-B/AChE/BuChE, AChE/BuChE, and H_3_-R/HMT/AChE/BuChE [21–24]. Structure-based approaches such as docking and molecular dynamics calculations have also been employed for the discovery of new multi-target ligands such as BuChE inhibitors/hCB2R and MAO-A/MAO-B/AChE/BuChE ligands to treat neurodegenerative diseases [25].

In this work, we offer a computational strategy for designing synthetically feasible ligands that bind to A_1_R and A_2A_R, and inhibit PDE10A—a novel multi-target combination of G protein-coupled receptors (GPCRs) and an enzyme, which has not, to our knowledge, been previously exploited. The designed ligands with this multi-target combination are intended as starting points for future development of multi-target drugs treating neurodegenerative diseases. It should be noted here that in the current study we only consider affinity of ligands to the above receptors, which we also experimentally validate as outlined below. However, for therapeutically relevant purposes also functional effects and optimization of selectivity towards A_1_R, A_2A_R and PDE10A need to be considered, which will be the area of a future study.

**Fig. 1:**
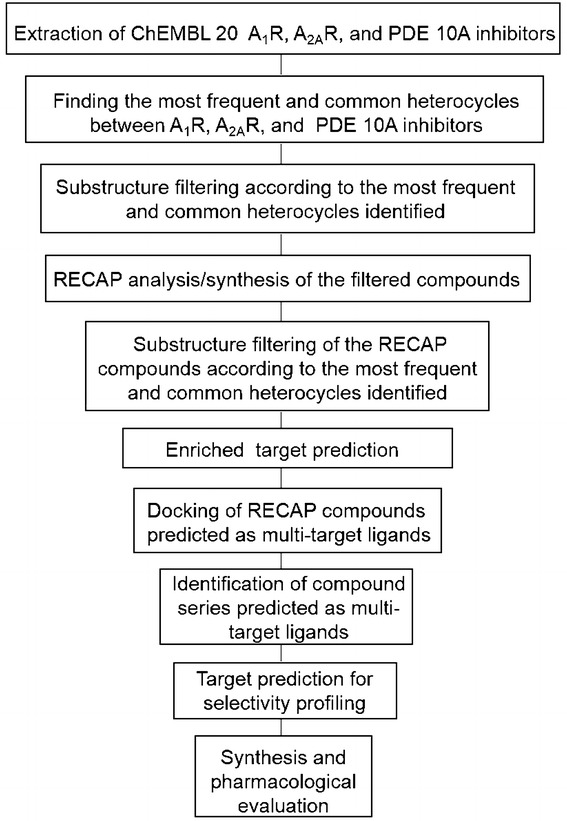
The computational strategy for rational design of A_1_R/A_2A_R–PDE10A multi-target ligands started with a focused chemical space consisting of known actives of A_1_R, A_2A_R and PDE10A, and formed new synthetically feasible compounds which were subjected to target prediction and docking for synthesis and pharmacological evaluation

The workflow of the current study is shown in Fig. 1. Starting with a focused chemical space consisting of known actives against A_1_R, A_2A_R and PDE10A, new synthetically feasible compounds were established via RECAP (Retrosynthetic Combinatorial Analysis Procedure) [26, 27], which fragments molecules at pre-defined bonds and recombines them in a combinatorial manner, and were then evaluated in silico, using target prediction and ligand/protein docking. Compounds with favorable assessments in both steps were carried forward for substructural analysis. This analysis identified compound series with the highest frequency of prediction as multi-target ligands against the desired set of targets, which is of advantage from the practical side, given their synthetic accessibility via a common synthetic route.

 A series of 2-aminopyridine-3-carbonitriles were selected for prospective validation of the pipeline, a series which was synthetically accessible via a one pot synthetic scheme i.e. providing products with the desired properties: cost-effective, synthetically efficient and available in a timely fashion [28, 29].

Subsequently the synthesized compounds were experimentally tested and confirmed as A_1_R/A_2A_R–PDE10A multi-target ligands. Selectivity against other subtypes of both protein families confirmed the pharmacological profile of the compound series, and structure activity relationships (SAR) were also deduced. Hence, in this work we report a successful computational strategy, which allowed the discovery of the first A_1_R/A_2A_R–PDE10A multi-target ligands. The novel A_1_R/A_2A_R–PDE10A ligands are sought to display a combination effect in modulating the A_1_R, A_2A_R, and PDE10A targets simultaneously similar to that of combination compounds of Adenosine receptors and PDEs, reported by Rickles et al., which were synergistic in modulating cAMP levels [10].

## Results and discussion

### Design of synthetically feasible A_1_R/A_2A_R–PDE10A multi-target ligands

Human enzyme and receptor data were extracted from ChEMBL 20 [30]. Substructure analysis of A_1_R, A_2A_R ligands and PDE10A inhibitors with K_i_ and IC_50_ values less than or equal to 1 µM revealed that the most frequently occurring common heterocycles among the actives against the three target classes were pyridine, pyrimidine, piperazine, and 1H-pyrazole (Additional file 1: Figure S1). Subsequently, A_1_R (2104), A_2A_R (2489) and PDE10A inhibitors (679) containing those frequent heterocycles were subjected to RECAP analysis/synthesis in MOE (see Methods for details) [26]. As a result, 458,839 (potentially) synthetically accessible ligands were formed in silico. This list of candidates was filtered to those retaining the common heterocycles (listed above), in order to create a focused chemical space characteristic of A_1_R, A_2A_R and PDE10A (with the simultaneous trade-off of reduced novelty), giving rise to 22,233 compounds.

### Target prediction of the designed RECAP library

To assess the likelihood of active compounds against A_1_R, A_2A_R and PDE10A, PIDGIN 1.0 (Prediction including Inactivity), a tool which uses ECFP 4 circular Morgan fingerprints and trained on ChEMBL actives and PubChem inactives, was used to perform in silico target prediction for the focused RECAP library (22,233 compounds) [24]. Subsequent enrichment analysis of the predictions was done using an estimation score, average ratio as developed by Liggi et al. [31] and via Chi square test [32]. For targets to be considered as enriched according to these methods, the estimation score and the Chi square test *p* value should be less than or equal to 0.01 and 0.05, respectively. Hence, upon analyzing the enrichment parameters for the A_1_R, A_2A_R and PDE10A targets that were predicted for the focused RECAP library (Additional file 1: Figure S2), the three targets were predicted with an estimation score equal to 0 (enriched) as well as average ratios less than 0.1 (enriched) with Chi squared *p* values < 0.005. The percentage of RECAP compounds of the focused library that were predicted as actives against the A_1_R, A_2A_R and PDE10A targets were 51.1, 52.8, and 24.5% respectively. These numbers are relatively high, which however is understandable given that the input to the RECAP analysis consisted of experimentally established known ligands of the above protein targets.

### Docking of the compounds predicted as A_1_R/A_2A_R–PDE10A multi-target ligands

In the next step docking and further substructure analysis were performed on compounds of the focused RECAP library, which were predicted as A_1_R/A_2A_R–PDE10A multi-target ligands from the ligand-based side in the previous step. 2563 compounds were predicted as actives against the three desired targets, and they were subsequently docked against a high resolution (1.8 Å) A_2A_R protein crystal structure (PDB ID: 4EIY) [33] its corresponding A_1_R homology model (see Methods for details), and PDE10A (PDB ID: 4DDL) [34].

Compounds which were carried forward to substructural analysis were selected when their docking score gave a value less than a pre-determined cut-off value computed from the docking scores. This cut-off value was evaluated as the docking score with the best F measure statistic obtained by docking a set of known actives and inactives against the protein crystal structures and the homology model (see Methods for details).

As a result, a distribution of RECAP compounds that were favorable as multi-target ligands by target prediction and docking was obtained, where 62.47% of the RECAP compounds that were predicted as A_1_R/A_2A_R–PDE10A multi-target ligands and docked against PDE10A exhibited docking scores lower than − 6.49 (the threshold of the best F measure discriminating between actives and inactives for known ligands). Out of the RECAP compounds which displayed docking scores lower than − 6.49 against PDE10A, 48.89 and 35.23% displayed docking scores lower than − 7.26 and − 8.49 against A_1_R and A_2A_R (the thresholds of the best F measures).

### Substructure analysis of the compounds predicted as A_1_R/A_2A_R–PDE10A multi-target ligands

**Fig. 2:**
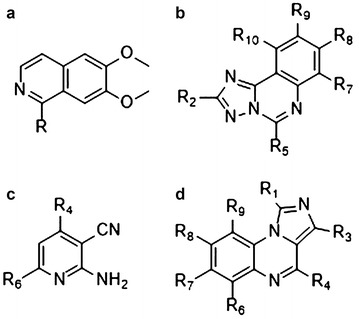
2563 compounds of the focused RECAP library were predicted as A_1_R/A_2A_R–PDE10A multi-target ligands, and docked against the A_2A_R protein crystal structure (PDB ID: 4EIY), A_1_R homology model, and the PDE10A protein crystal structure (PDB IB: 4DDL), the RECAP series which showed an agreement between the ligand-based and structure-based predictions were mainly **a** 6,7-alkoxyisoquinolines **b** [1,2,4] triazolo[1,5-c]quinazolines **c** 2-aminopyridine-3-carbonitriles **d** imidazo[1,5-a]quinoxalines

Substructure analysis was performed on compounds having a favorable assessment by target prediction and docking (i.e. those compounds whose docking scores were below the threshold for all three targets). The analysis revealed frequently occurring series, which shared the same core structure and which are shown in Fig. 2.

The chemical series were identified as [1,2,4]triazolo[1,5-c]quinazolines (50.4% of all positively predicted multi-target ligands by in silico target prediction as well as docking), imidazo[1,5-a]quinoxalines (14.4%), 6,7-alkoxyisoquinolines (10.6%), and 2-aminopyridine-3-carbonitriles (9.2%). These were in addition to various compounds containing the common and frequent heterocycles identified earlier (15.4%). Each series identified could be considered for synthesis, SAR studies and validation as A_1_R/A_2A_R–PDE10A multi-target ligands.

### Synthesis of novel 2-aminopyridine-3-carbonitriles

**Scheme 1:**
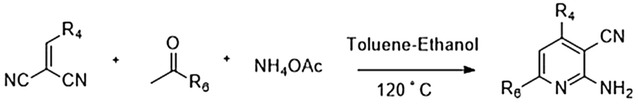
The one-pot synthetic route followed for the synthesis of novel 4,6-substituted 2-amino-pyridin-3-carbonitriles

Due to both ease of the reaction and anticipated yield, a one-pot synthetic scheme was selected for synthesizing one promising series, 2-aminopyridine-3-carbonitriles. The design resulted in 25 compounds for synthesis of which 21 were novel compounds and four (**1**, **2**, **5**, and **17**) have previously been reported in the literature [35–38]. Compounds **1**–**25** were screened against PAINs (PAN Assay Interference Compounds) [39] using FAFDrug3 [40], and none of the compounds exihibited potential PAINs liability. Subsequently, their synthesis was performed as shown in Scheme 1, and all products were obtained with good yields, ranging from 46 to 85% (see Methods for details).

### Pharmacological evaluation of novel 2-aminopyridine-3-carbonitriles

Bioactivity testing was performed using A_1_ and A_2A_ human adenosine receptors expressed in transfected CHO (A_1_) and HeLa (A_2A_) cells, as well as AD293 cells that were transiently transfected with human PDE10A. Table 1 includes the list of synthesized 4,6-substituted 2-amino-pyridin-3-carbonitriles, along with their K_i_ values against A_1_R, A_2A_R, and IC_50_ values against PDE10A. It can be seen that 15 compounds of the 25 synthesized 2-amino-pyridin-3-carbonitriles exhibited inhibitory activity against PDE10A below 10 μM. In addition, 13 compounds were adenosine receptor binders exhibiting selectivity towards A_1_R and A_2A_R, which has not been the case in the previous work reported by Mantri et al., where 2-amino-pyridin-3-carbonitriles were promiscuous towards the four adenosine receptor subtypes [36].

**Table 1:**
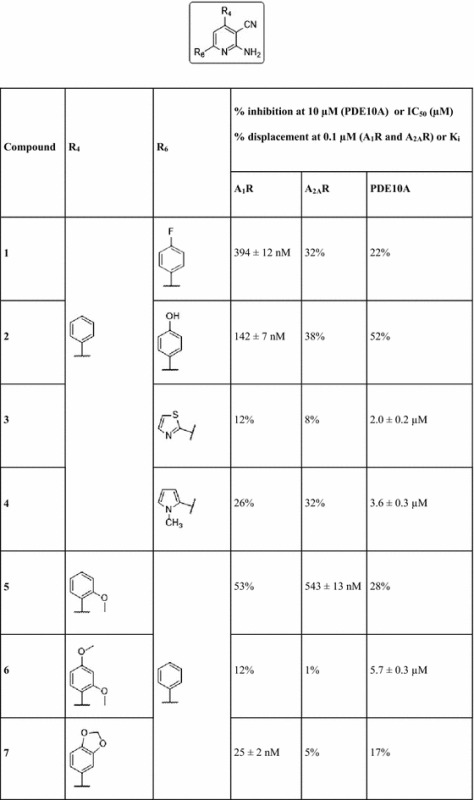

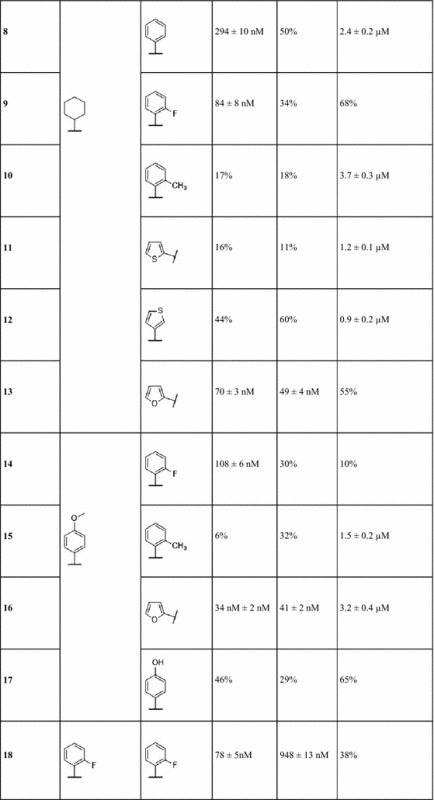

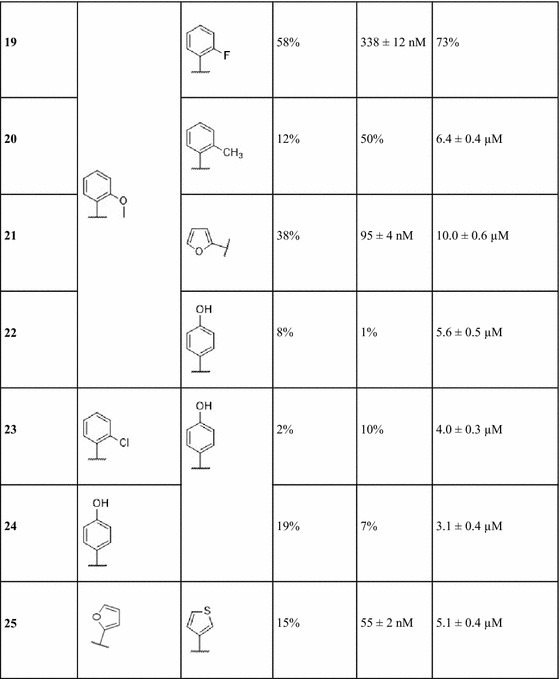
Percent inhibition of the synthesized 4,6-substituted 2-amino-pyridin-3-carbonitriles at 10 µM (PDE10A) or IC_50_ (µM) and percentage displacement at 0.1 µM (A_1_R and A_2A_R), or K_i_

IC_50_ values of the 2-aminopyridines-3-carbonitriles were measured for the four phosphodiesterases PDE7A, PDE7B, PDE9A and PDE10A at 10 μM concentration. For those compounds that showed percentage inhibition greater than 70% and selectivity against other measured isoenzymes, IC_50_ were determined. Calculation of the K_i_ values at A_1_R, A_2A_R, A_2B_R and A_3_R was approximated using the Cheng-Prusoff equation: K_i_ = IC_50_/[1 + (C/K_D_)], where IC_50_ is the concentration of compound that displaces the binding of the radioligand by 50%, C is the concentration of radioligand, and K_D_ is the dissociation constant of each radioligand

Given that the objective of this work is to find compounds displaying specific multi-target activity, compounds **8**, **16**, **21**, and **25** were identified as A_1_R/A_2A_R–PDE10A multi-target ligands, inhibiting PDE10A with IC_50_ values of 2.4, 3.2, 10.0, and 5.1 µM respectively, and binding to A_1_R with K_i_ values of 294 and 34 nM (compounds **8** and **16**, respectively), and to A_2A_R with K_i_ values of 41, 95, and 55 nM (compounds **16**, **21**, and **25**, respectively). Notably, compound **16** exhibited the desired multi-target profile as a PDE10A inhibitor and a dual binder to A_2A_R and A_1_R.

It was previously reported that substituted pyridines exhibited PDE inhibitory activity [41, 42], and 2-amino-pyridin-3-carbonitriles are adenosine receptor ligands [36]. In this study we have now identified suitable compounds matching *both* criteria as A_1_R/A_2A_R–PDE10A multi-target ligands, satisfying the original compound design objective.

### (SAR) structure–activity relationship analysis

The purpose of the SAR analysis was to rationalize the variation in activity of the newly discovered A_1_R/A_2A_R–PDE10A multi-target ligands against PDE10A, given that 2-amino-pyridin-3-carbonitriles have been discovered as a novel class of PDE10A inhibitors. Also due to the fact that compounds of this substructural class were documented as adenosine receptor ligands [36], computational SAR studies were focused on the PDE10A data, where the variation in potency was rationalized in relation to the physicochemical properties of the compounds (which were computed by FAFDrug3, Additional file 1: Table S1) [40].

A trend observed repeatedly in several cases was that when logP decreased, associated with an increase in tPSA, then this led to an improvement in the activity against PDE10A. Initial analysis concentrated on compounds **1–4**, which have a phenyl substituent at position 4 of the pyridine ring. Compound **3** was the most potent PDE10A inhibitor with an IC_50_ of 2.0 µM, and a computed logP of 3.1 and tPSA of 103.9 Å^2^. Similarly, for compounds **5**–**7** having a phenyl substituent at position 6 of the pyridine ring, compound **6** was the most potent against PDE10A with an IC_50_ of 5.7 µM and a computed logP of 4.0 and tPSA of 81.2 Å^2^. For compounds **8**–**13**, which have a cyclohexyl ring at position 4 of the pyridine ring, compound **12** displayed the most potent PDE10A inhibitory activity with an IC_50_ of 0.9 µM and a computed logP of 4.7 and tPSA of 90.9 Å^2^. For compounds **14**–**17**, with a p-methoxyphenyl substituent at position 4 of the pyridine ring, compound **16** with the smallest predicted lipophilicity of 3.1 and tPSA of 85.1 Å^2^ displayed a good PDE10A inhibitory activity with an IC_50_ value equal to 3.2 µM, yet the most potent compound was **15** with an IC_50_ value of 1.5 µM and a computed logP of 4.4 and tPSA of 71.9 Å^2^. For compounds **19**–**22**, with an o-methoxyphenyl substituent at position 4 of the pyridine ring, compound **22** displayed PDE10A inhibitory activity with the highest potency (IC_50_ value of 5.6 µM), and a computed logP of 3.7 and tPSA of 92.2 Å^2^. Finally a similar general trend is observed for the compounds **23** and **24** with a 4-hydroxyphenyl substituent at position 6 of the pyridine ring, where compound **24** was a more potent PDE10A inhibitor with an IC_50_ of 3.1 µM and computed logP of 3.4 and tPSA of 103.2 Å^2^. Hence, it could be deduced that in the majority of the series considered, where the substituents on a single position is varied, a decrease in computed lipophilicity associated with an increase in polarity generally improved the activity of compounds against PDE10A. This general trend can be attributed to the hydrophilic nature of the pocket, which favours the interactions between the ligand and the PDE10A protein by compounds exhibiting these properties.

### Compound selectivity assessment

The selectivity of compounds **1**–**25** against the selected major off-targets A_2B_R, A_3_R, PDE7A, PDE7B, and PDE9A, was predicted using PIDGIN at a threshold for binding greater than or equal to 0.8, and subsequently tested experimentally. It is noted here that the IC_50_ values were determined for compounds with % inhibition at phosphodiesterases greater than 70%. As shown in Additional file 1: Table S2, the synthesized compounds are mostly inactive against those off-targets except for compounds **16**, **17**, **21**, and **23** that exhibited IC_50_ values of 3.4, 3.5, 15.1 and 1.8 µM against PDE7A, and compounds **23** and **25**, which exhibited IC_50_ values of 7.3 and 4.7 µM against PDE7B. Remarkably, compound **8** was found to exhibit selectivity over all tested off-targets using the above criterion, with the lowest selectivity measured for PDE7B (of 55% inhibition at 10 µM ligand concentration). This can be compared to the IC_50_ value of **8** at PDE10A, which is 2.4 μM (indicating approximately twofold selectivity for **8**).

In general, the experimental results on off-target prediction for the synthesised 4,6-substituted 2-amino-pyridin-3-carbonitriles **1**–**25** agree with the predictions generated using PIDGIN utilised to bias the compound design towards selective compounds such as **8** (Additional file 1: Table S2). This compound would serve as a good starting point for analog modification to improve the selectivity of the synthesized ligands towards PDE10A.

### Analysis of the molecular docking studies of the synthesized 2-aminopyridine-3-carbonitriles

**Fig. 3:**
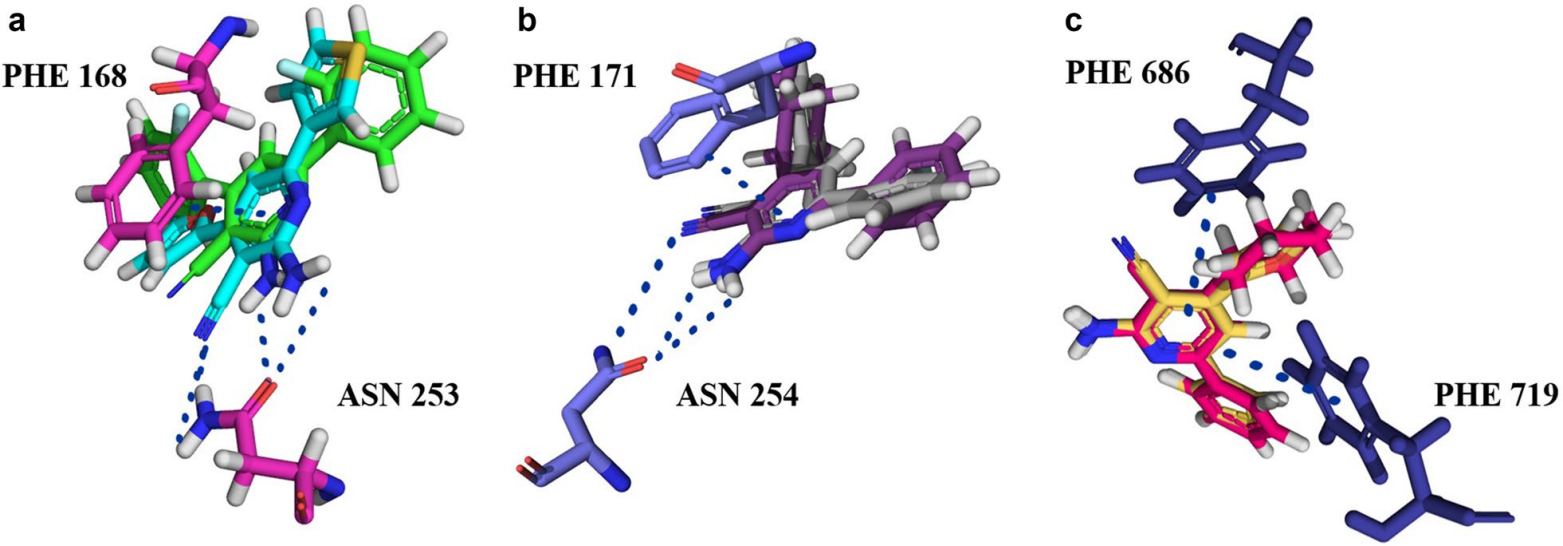
Docking studies predicted molecular interactions characteristic of the 4,6-substituted 2-amino-pyridin-3-carbonitriles with the A_2A_R protein crystal structure (PDB ID: 4EIY), A_1_R homology model, and PDE10A protein crystal structure (PDB ID: 4DDL), which are displayed for representative multi-target ligands with the following combinations: compound **8** (A_1_R–PDE10A), **18** (A_1_R–A_2A_R), and **25** (A_2A_R–PDE10A): **a** interactions with A_2A_R: the overlaid compounds **18** and **25** exhibit H-bonds via amino and carbonitrile groups with Asn_253_, and the pyridine rings are π-stacked with Phe_168_ **b** interactions with A_1_R: the overlaid compounds **8** and **18** exhibit H-bonds via amino and carbonitrile groups with Asn_254_, and the pyridine rings are π-stacked with Phe_171_ **c** interactions with PDE10A: the overlaid compounds **8** and **25** have the pyridine rings π-stacked with Phe_686_ and Phe_719_. The molecular interactions predicted for the active molecules are consistent with observed interactions between co-crystallised ligands and their corresponding protein crystal structures (PDB ID: 4EIY and 4DDL) [33, 34] and the interactions with the A_1_R homology model reported in the literature [51, 52]

The synthesized 2-aminopyridine-3-carbonitriles were docked against A_2A_R (PDB ID: 4EIY), A_1_R homology model, and PDE10A (PDB ID: 4DDL). Figure 3 shows the common predicted ligand-target interactions for representative multi-target ligands of A_1_R–PDE10A, A_1_R–A_2A_R, and A_2A_R–PDE10A, namely for compounds **8**, **18**, and **25**.

It can be seen that compounds **8** and **25**, with IC_50_ values of 2.4 and 5.1 µM respectively, share similarities in predicted binding modes, since their pyridine rings display π-stacking with Phe_686_ and Phe_719_ of PDE10A (Fig. 3). These are the type of interactions predicted to be exhibited by the majority of the synthesized ligands from this work, as well as the only existing interactions between co-crystallised PDE10A inhibitors discovered by fragment screening (PDB ID: 5C2E, 5C1W, 5C29, 5C2A ligands with K_i_ values of 2, 8, 700, 880, and 4.8 nM, respectively) [43]. It is noted that the ligand of 5C2A exhibits a considerable selectivity towards PDE10A over all the other PDEs (in the range of 100–1000 fold and greater over the majority of PDEs, with the least selectivity observed being in the range of 25–100 fold). This ligand exhibits only π-stacking interactions with Phe_686_ and Phe_719_, similar to the mode of interactions of compound **8** with PDE10A, which is relatively selective over all tested PDEs, with the lowest selectivity being measured for PDE7B (of 55% inhibition at 10 µM ligand concentration) and compound **25**, which is selective against all tested PDEs except PDE7B (Table 1 and Additional file 1: Table S2). Additional interactions were seen in analogs discovered by fragment screening, namely hydrogen bonding with Gln_716_ and Tyr_683_ in the PDE10A selectivity pocket (PDB ID: 5C28 and 5C2H with K_i_ values of 2200 and 0.0082 nM respectively). [43] The ligand of 5C2H exhibits π-stacking with Phe_686_ and Phe_719_ and hydrogen bonding with Tyr_683_ in the PDE10A selectivity pocket. The 5C2H ligand showed a very high selectivity towards PDE10A, greater than 5000 fold, which emphasizes the consideration of compound **8** for analog modification to target the selectivity pocket in order to improve the folds of selectivity towards PDE10A. In addition, hydrogen bonding with Tyr_683_ in the PDE10A selectivity pocket is also seen in many other highly selective PDE10A inhibitors reported in the literature [44] (PDB ID: 5DH5, [45] 5B4L, [46] with K_i_ = 0.23 nM, and IC_50_ = 0.76 nM respectively), which further highlights the importance of analog modification to target the PDE10A selectivity pocket.

Moreover, it is noted that compounds **16** and **21** with IC_50_ values of 3.2 and 10.0 µM respectively (which are selective against all tested PDEs except PDE7A, Table 1 and Additional file 1: Table S2) were predicted to exhibit an additional type of interaction, H-bonding with Gln_716_ via their overlaid furan rings at position 6 of the pyridine ring (Additional file 1: Figure S3). In fact H-bonding with Gln_716_ was the only interaction, besides π-stacking with Phe_686_ and Phe_719_, which has been observed in many of the highly selective PDE10A ligands reported in the literature (PDB ID: 4DDL, [34] 3SN7, 3SNL, and 3SNI, [47] 5DH4 and 5DH5, [45] with IC_50_ values of 4.9, 0.7, 0.7, 11 nM and K_i_ = 0.23 nM respectively). As for other type of interactions generally exhibited by known PDE10A inhibitors such as hydrogen bonding with Gln_726_ and π-stacking with Phe_729_ (PDB ID: 5EDE) [48], none has been predicted for any of the compounds presented in this work.

Common predicted binding modes can also be observed for the synthesized compounds against the adenosine receptors A_2A_R and A_1_R. Figure 3 displays the interactions of two representative compounds **18** and **25**, which exhibit K_i_ values of 948 and 55 nM respectively, and these are H-bonding of their pyridine rings with Asn_253_ and π-stacking of their amino and carbonitrile groups with Phe_168_ of A_2A_R. As for A_1_R, the overlaid compounds **8** and **18**, with K_i_ values of 294 and 78 nM respectively, H-bond via their amino and carbonitrile groups with Asn_254_, and their pyridine rings are π-stacked against Phe_171_. It can be observed that the ligand/protein interactions predicted for the active compounds against the A_2A_R are also those seen in the co-crystallised ligand/protein crystal structures (PDB ID: 4EIY, [33] 3EML, [49] 5IU4, [50] with a K_i_ value of 0.8 nM for ZM241385, which is the common ligand for the three PDB IDs). Similar was the case for the reported interactions with the A_1_R homology model in the literature (with IC_50_ values of 2.9 and 6.2 nM for the reported ligands predicted to bind to the homology model of A_1_R) [51, 52].

Generally the compounds exhibited good selectivity towards A_1_R and A_2A_R (Table 1 and Additional file 1: Table S2) with a nanomolar range of binding affinities. As for the selectivity towards PDE10A, it could be improved by analog modification of compound **8**, which favors the hydrogen bonding with Tyr_683_ in the PDE10A selectivity pocket. In addition, the potency of compounds against PDE10A could be optimized in itself, in order to achieve therapeutically relevant efficacy.

### Computational assessment of CNS permeability

Compounds **8** and **16** exhibited the desired multi-target profile by inhibiting PDE10A and binding to A_2A_R and/or A_1_R. The physicochemical properties of these compounds were calculated by FAFDrug3 [40], and both compounds passed the Lipinski rule of 5 and the CNS filter, which takes into consideration the assessment of their ability to pass the blood brain barrier (Additional file 1: Figure S4) [53]. Hence, while further experimental work would be needed to establish the validity of those predictions, compounds **8** and **16** may serve as good starting points for further functional efficacy assessment and selectivity optimization towards PDE10A, A_2A_R and/or A_1_R for the subsequent consideration of multi-target drug development for the treatment of neurodegenerative diseases.

## Conclusions

Here we report a successful computational strategy for designing the first A_1_R/A_2A_R–PDE10A multi-target ligands as a therapeutic prospect for neurodegenerative diseases. A retrosynthetic approach was employed using MOE/RECAP, followed by target prediction and docking of the resulting library against the desired targets. We have identified 2-aminopyridine-3-carbonitriles as a series that showed agreement between both the ligand- and structure-based predictions of activity against A_1_R, A_2A_R and PDE10A. The synthesis of this series via a one-pot synthetic scheme was pursued experimentally. As a result, compounds **8**, **16**, **21**, and **25** were validated as A_1_R/A_2A_R–PDE10A multi-target ligands with IC_50_ values of 2.4, 3.2, 10.0, and 5.1 µM against PDE10A, and binding to A_1_R with K_i_ values of 294 and 34 nM (**8** and **16** respectively), and to A_2A_R with K_i_ values of 41, 95, and 55 nM (**16**, **21**, and **25** respectively). Furthermore, selectivity profiling of the synthesized 4,6-substituted 2-amino-pyridin-3-carbonitriles against other subtypes of both protein families showed that the multi-target ligand **8** exhibited a minimum of twofold selectivity over all tested off-targets. In addition, compounds **8** and **16** exhibited the desired multi-target profile against A_1_R, A_2A_R and PDE10A, which would serve as good starting points for further functional efficacy assessment and analog modification for the improvement of selectivity. In particular, this comprises investigating the signal transduction profiles of these compounds using techniques some of the authors have described before [51], as well as evaluating functional effects in cAMP assays to determine if these compounds do provide synergistic elevations in intracellular cAMP. One specific functional profile that would be of high interest and which is likely to elevate cAMP levels synergistically via the combination effect on multiple targets simultaneously, is the A_1_R antagonist/A_2A_R agonist, and PDE10A inhibitor.

In summary we have investigated a computational approach for the design of multi-target ligands that was validated experimentally via synthesis and pharmacological evaluation of 2-aminopyridine-3-carbonitriles as A_1_R/A_2A_R–PDE10A ligands. This approach is generally applicable to a wide range of multi-target ligand design problems, across disease areas and target families.

## Experimental

### Selecting reference molecules for the design of multi-target ligands

Using SQL (script provided in Additional file 1), human A_1_R (2860), A_2A_R (3566) ligands and PDE10A inhibitors (843) were extracted from the ChEMBL 20 database with K_i_ and IC_50_ values less than or equal to 1 μM respectively, and confidence scores of 8 or 9 [30]. Following extraction, the most frequent and common heterocycles between A_1_, A_2A_ receptor ligands and PDE10A inhibitors were found by performing substructure analysis on each structure using the “Chemistry-> Analyze scaffolds” function in DataWarrior 4.2.2 [54]. Analysis of A_1_R, A_2A_R ligands and PDE10A inhibitors identified common and frequent heterocycles (pyridine, 1H-pyrazole, pyrimidine and 9H-purine for A_1_R and A_2A_R), and these were extracted from each set using RDKit, 9.1, Python [55]. It should be noted that compounds containing 9H-purine were also extracted from the original set even though this substructure is characteristic of A_1_R and A_2A_R only, since it is structurally similar to the common and frequent heterocycles identified (pyridine, 1H-pyrazole, and pyrimidine). Additional file 1: Figure S1 shows the most frequent heterocycles for the A_1_R, A_2A_R ligands, and PDE10A inhibitors and their relative frequencies in each set. It was found that they are furan, pyridine, xanthine, 1H-pyrazole, pyrimidine, piperazine, and 9H-purine. All of these heterocycles ranked among the top 30 for A_1_R, A_2A_R ligands and PDE10A inhibitors. This indicated their suitability for designing multi-target ligands at these protein targets, given the overlap in chemical (heterocyclic) space. In the case where no percentage is displayed for a particular target, this means that the heterocycle does not appear among the top 30 for the set of compounds involved.

### Designing new multi-target ligands

A_1_R (2104), A_2A_R (2489) and PDE10A inhibitors (679) consisting of the common and frequent heterocycles, were subjected to RECAP analysis/synthesis in MOE [26]. The RECAP function electronically fragments and recombines molecules based on chemical knowledge of 11 chemical bond types derived from common chemical reactions [27]. As a result, 458,839 novel RECAP-derived compounds were formed. Finally the designed RECAP library was filtered using RDKit, Python according to the common and frequent heterocycles identified, which narrowed the list down to 22,233 compounds.

### Target prediction

The SMILES of the designed RECAP library were standardized using the ChemAxon Command-Line Standardizer where the following options were selected: “Remove Fragment” (keep largest), “Neutralize”, “RemoveExplicitH”, “Clean2D”, “Mesomerize” and “Tautomerize” [56]. The standardized canonical SMILES were exported to CSV files, and subjected to enriched target prediction using PIDGIN 1.0 implementing the method developed by Liggi et al. [24, 31]. The target prediction for the designed RECAP library was performed using a recall probability threshold of 0.01 (which is a value consistent with greater confidence in the more positive predictions).

Enrichment calculations for the predicted targets of the designed RECAP library were performed to assess the likelihood of the active compounds against the targets of interest. In this procedure, the frequency of predicting A_1_R, A_2A_R and PDE10A targets for the designed RECAP library was compared with a background distribution of a diverse library covering a large chemical space and was assessed by two parameters: the estimation score and the average ratio. The cutoff selected for considering a target as sufficiently enriched required an estimation score less than or equal to 0.01 [31]. The statistical relevance of the prediction was assessed via a Chi squared test with yates correction in Scipy [32], using the contingency table of the RECAP library and background of randomly sampled PubChem compounds (Additional file 1: Figure S2).

### Receptor preparation

Docking with Glide [57] was performed against the human A_2A_R protein crystal structure (PDB ID: 4EIY) bound to the antagonist ZM241385 and the PDE10A crystal structure (PDB ID: 4DDL) complexed with an inhibitor [33, 34]. Protein structures were prepared using the protein preparation wizard of maestro 9.3 [58], following the default protocol which accounts for energy refinement, hydrogen addition, pKa assignment, and side-chain rotational isomer refinement. Resolved water molecules were discarded, and the structure was centered using the co-crystallized ligand as the center of the receptor grid generated for each protein structure. The co-crystal structures of A_2A_R with 4-{2-[(7-amino-2-furan-2-yl[1, 2, 4]triazolo[1,5-a][1, 3, 5]triazin-5-yl)amino]ethyl}phenol (PDB ID: 4EIY), and PDE10A with 2-{1-[5-(6,7-dimethoxycinnolin-4-yl)-3-methylpyridin- 2-yl]piperidin-4-yl}propan-2-ol (PDB ID: 4DDL), were selected as target structures.

The A_1R_ homology model (Additional file 2) was constructed according to the method reported by Yaziji et al. [59–61], where the protein sequence of the human A_1_R (accession number P30542) was aligned with the A_2A_R template of PDB ID: 4EIY.

### Ligand preparation

The entire set of 2563 ligands was prepared for docking with LigPrep 2.5 [62] using the default settings and the Epik option which introduces energy penalties associated with ionization and tautomerization [63].

### Cut-off generation for compound selection from docking models

In an attempt to validate the constructed A_2A_R, A_1_R, and PDE10A docking models, a set of known actives and inactives were docked against each target to ensure that they enriched actives. 81 A_2A_R receptor ligands reported in the literature were docked against the A_2A_R model [64, 65]. For consistency 81 ChEMBL actives were also selected (for each of the A_1_R and PDE10A proteins whose K_i_ and IC_50_ values are less than 10 µM), and these were docked against their respective target class. In addition, PubChem inactives (200 compounds) of each target class were docked.

A good separation was obtained for the medians of docking score distribution for actives versus inactives confirming that the actives are enriched. Additional file 1: Figure S5 shows the separation of the medians for the three docking models, − 6.93 (actives) versus − 5.64 (inactives) for the PDE10A docking model, − 7.66 (actives) versus − 6.01 (inactives) for the A_2A_R docking model, and − 7.60 (actives) versus − 5.66 (inactives) for the A_1_R docking model. Statistical analysis was performed with R using a Mann–Whitney test [66] on the active and inactive docking score distributions of each target. The differences in medians were significant with *p* values < 0.05 (script provided in Additional file 1).

The F_1_ score which is the harmonic mean of precision and recall, was computed (using a Python script, see Additional file 1) for all the docking scores of the ChEMBL actives and PubChem inactives for each model. A search was performed for a docking score threshold that gave the highest F_1_ score, in order to perform substructure analysis on compounds that were predicted as A_1_R/A_2A_R–PDE10A multi-target ligands by target prediction, and displayed docking scores that are lower than or equal to those with the highest F_1_ score for each of the three docking models (A_1_R, A_2A_R, and PDE10A, see Additional files 3, 4, and 5). Furthermore, the thresholds found are intended to serve as reference scores for any structure-based design problem at these target classes.

### Docking

The RECAP compounds that were predicted as A_1_R/A_2A_R–PDE10A multi-target ligands were docked against the A_2A_R protein crystal structure (PDB ID: 4EIY) [33], the A_1_R homology model and the PDE10A protein crystal structure (PDB ID: 4DDL) [34] to investigate the molecular interactions. The Glide docking parameters used here are given in Additional file 1: Table S3. The parameters were deduced from docking experiments using known actives and inactives against each protein model.

### Substructural analysis

Subsequently, substructure analysis was performed using DataWarrior 4.2.2, on the proposed A_1_R/A_2A_R–PDE10A multi-target ligands predicted by both ligand-based and structure-based techniques (considering docking scores less than or equal to the threshold of the best F measure for each docking model). The chemical series found were [1,2,4] triazolo[1,5-c]quinazolines (50.4%), imidazo[1,5-a]quinoxalines (14.4%), 6,7-alkoxyisoquinolines (10.6%), and 2-aminopyridine-3-carbonitriles (9.2%), in addition to various compounds consisting of the common and frequent heterocycles identified originally in the substructural analysis of the extracted ChEMBL compounds.

### Synthesis of novel 4,6-substituted 2-amino-pyridin-3-carbonitriles

Due to both ease of the reaction and yield, a one-pot synthetic scheme was optimized for the purpose of synthesizing 2-aminopyridine-3-carbonitriles. For the other series, the synthetic routes were multi-step reactions, which due to synthetic complexity are not reported here.

The synthetic routes reported in the literature for the formation of derivatives of 6,7-alkoxyisoquinolines as selective PDE10A inhibitors involved multi-step reactions ranging from 3 to 13 steps [67, 68]. Whereas, the procedures for the synthesis of the imidazo[1,5-a]quinoxalines, known PDE10A inhibitors, consisted of 3–7 step reactions [69–72]. The [1,2,4]triazolo[1,5-c]quinazolines have been reported as potent and selective A_2A_R antagonists and PDE10A inhibitors, and their synthesis involved 4–7 step reactions [73–75].

Hence, given the fact that the 2-aminopyridine-3-carbonitriles were the only RECAP series that could be synthesized via a one-pot synthetic scheme [37, 76, 77], we have selected these for synthesis and subsequent validation as multi-target ligands. In particular, we selected compounds, which did not exihibit any potential PAINs liability upon screening with the FAFDrug3 ADME-Tox Filtering Tool [40].

#### Chemistry

Unless otherwise indicated, all starting materials, reagents and solvents were purchased and used without further purification. After extraction from aqueous phases, the organic solvents were dried over anhydrous sodium sulfate. The reactions were monitored by thin-layer chromatography (TLC) on 2.5 mm Merck silica gel GF 254 strips, and each of the purified compounds showed a single spot; unless stated otherwise, UV light and/or iodine vapor were used to detect compounds. The synthesis of the target compounds was performed in coated Kimble vials on a PLS (6 × 4) Organic Synthesizer with orbital stirring. Filtration and washing protocols for supported reagents were performed in a 12-channel vacuum manifold. The purity and identity of all tested compounds were established by a combination of HPLC, elemental analysis, mass spectrometry and NMR spectroscopy as described below. Purification of isolated products was carried out by column chromatography (Kieselgel 0.040–0.063 mm, E. Merck) or medium pressure liquid chromatography (MPLC) on a CombiFlash Companion (Teledyne ISCO) with RediSep pre-packed normal-phase silica gel (35–60 µm) columns followed by recrystallization. Melting points were determined on a Gallenkamp melting point apparatus and are uncorrected. The NMR spectra were recorded on Bruker AM300 and XM500 spectrometers. Chemical shifts are given as δ values against tetramethylsilane as internal standard and J values are given in Hz. Mass spectra were obtained on a Varian MAT-711 instrument. Analytical HPLC was performed on an Agilent 1100 system using an Agilent Zorbax SB-Phenyl, 2.1 mm × 150 mm, 5 µm column with gradient elution using the mobile phases (A) H_2_O containing 0.1% CF_3_COOH and (B) MeCN and a flow rate of 1 mL/min. The purity of all tested compounds was determined to be greater than or equal to 95%.

The synthesis of the 4,6-substituted 2-amino-pyridin-3-carbonitriles **1**–**25** was done via the one-pot synthetic route shown in Scheme 1. Varying both substituents on the ylidene malononitrile and the ketone reagents resulted in a variation of the substituents on positions 4 and 6 of the pyridine ring.

### Synthetic procedure

Substituted ylidene malononitrile (1.0 mmol), ketone (1.0 mmol) and ammonium acetate (5.0 mmol) in a 1:1 toluene/EtOH mixture (7 mL) were stirred in a coated Kimble vial at 120 °C for 12–24 h. After reaction completion (TLC control), distilled water was added and the mixture was extracted with ethyl acetate (3 × 10 mL). The organic phase was dried (Na_2_SO_4_) and evaporated under reduced pressure to afford an oily residue that was purified by column chromatography using *n*-hexane-ethyl acetate in 2:1 mixture.

#### **2-amino-6-(4-fluorophenyl)-4-phenylpyridine-3-carbonitrile (1)**

Purified by column chromatography (*n*-hexane-ethyl acetate 2:1) and then recrystallized from EtOH to give 0.246 g, 85% yield (97% purity by HPLC). MP 226–228 °C. ^1^H NMR (300 MHz, CDCl3), δ (ppm) 8.08–7.95 (m, 2H), 7.69–7.58 (m, 2H), 7.60–7.47 (m, 3H), 7.23–7.09 (m, 3H), 5.34 (s, 2H). MS (EI) *m/z* (%): 289.07 (M^+^, 100), 262.07 (7). Analysis calculated for C_18_H_12_FN_3_: C, 74.73; H, 4.18; F, 6.57; N 14.52. Found: C, 74.70; H, 4.19; F, 6.55; N, 14.54.

#### **2-amino-6-(4-hydroxyphenyl)-4-phenylpyridine-3-carbonitrile (2)**

Purified by column chromatography (*n*-hexane-ethyl acetate 2:1) and then recrystallized from EtOH to give 0.227 g, 79% yield (96% purity by HPLC). MP 241–243 °C. ^1^H NMR (300 MHz, CDCl3), δ (ppm) 9.92 (s, 1H), 7.99 (d, J = 8.6 Hz, 2H), 7.78–7.59 (m, 2H), 7.58–7.47 (m, 3H), 7.15 (s, 1H), 6.88 (s, 2H), 6.83 (d, J = 8.7 Hz, 2H). MS (EI) *m/z* (%): 287.04 (M^+^, 100), 259.89 (10). Analysis calculated for C_18_H_13_N_3_O: C, 75.25; H, 4.56; N, 14.63; O, 5.57. Found: C, 75.27; H, 4.54; N, 14.62; O, 5.59.

#### **2-amino-4-phenyl-6-(1,3-thiazol-2-yl)pyridine-3-carbonitrile (3)**

Purified by column chromatography (*n*-hexane-ethyl acetate 2:1) and then recrystallized from EtOH to give 0.172 g, 62% yield (95% purity by HPLC). MP 154–156 °C. ^1^H NMR (300 MHz, CDCl3), δ (ppm) 7.95 (d, J = 3.0 Hz, 1H), 7.72 (s, 1H), 7.66–7.65 (m, 2H), 7.52–7.50 (m, 4H), 5.30 (s, 2H). MS (EI) *m/z* (%): 278.03 (M^+^, 100), 276.97 (45). Analysis calculated for C_15_H_10_N_4_S: C, 64.73; H, 3.62; N, 20.13; S, 11.52. Found: C, 64.85; H, 3.48; N, 20.25; S, 11.42.

#### **2-amino-6-(1-methyl-1H-pyrrol-2-yl)-4-phenylpyridine-3-carbonitrile (4)**

Purified by column chromatography (*n*-hexane-ethyl acetate 2:1) and then recrystallized from EtOH to give 0.189 g, 69% yield (98% purity by HPLC). MP 152–153 °C. ^1^H NMR (300 MHz, CDCl3), δ (ppm) 7.67–7.54 (m, 2H), 7.56–7.42 (m, 3H), 7.30 (s, 1H), 6.91 (s, 1H), 6.66–6.59 (m, 2H), 5.23 (s, 2H), 3.70 (s, 3H). MS (EI) *m/z* (%): 274.14 (M^+^, 100). Analysis calculated for C_17_H_14_N_4_: C, 74.43; H, 5.14; N, 20.42. Found: C, 74.57; H, 5.12; N, 20.30.

#### **2-amino-4-(2-methoxyphenyl)-6-phenylpyridine-3-carbonitrile (5)**

Purified by column chromatography (*n*-hexane-ethyl acetate 2:1) and then recrystallized from EtOH to give 0.238 g, 79% yield (97% purity by HPLC). MP 199–200 °C. ^1^H NMR (300 MHz, CDCl3), δ (ppm) 8.03–7.93 (m, 2H), 7.52–7.41 (m, 4H), 7.31 (dd, J1 = 7.5 Hz, J2 = 1.8 Hz, 1H), 7.17 (s, 1H), 7.11–7.02 (m, 2H), 5.27 (s, 2H), 3.88 (s, 3H). MS (EI) *m/z* (%): 301.16 (M^+^, 100), 270.12 (7), 120.10 (16.3). Analysis calculated for C_19_H_15_N_3_O: C, 75.73; H, 5.02; N, 13.94; O, 5.31. Found: C, 75.76; H, 5.04; N, 13.92; O, 5.33.

#### **2-amino-4-(2,4-dimethoxyphenyl)-6-phenylpyridine-3-carbonitrile (6)**

Purified by column chromatography (*n*-hexane–ethyl acetate 2:1) and then recrystallized from EtOH to give 0.238 g, 72% yield (99% purity by HPLC). MP 155–157 °C. ^1^H NMR (300 MHz, CDCl3), δ (ppm) 8.02-7.90 (m, 2H), 7.52–7.38 (m, 3H), 7.32–7.22 (m, 1H), 7.16 (s, 1H), 6.69–6.55 (m, 2H), 5.25 (s, 2H), 3.88 (s, 3H), 3.86 (s, 3H). MS (EI) *m/z* (%): 331.14 (M^+^, 100), 165.51 (9), 120.16 (11.3). Analysis calculated for C_20_H_17_N_3_O_2_: C, 72.49; H, 5.17; N, 12.68; O, 9.66. Found: C, 72.50; H, 5.19; N, 12.71; O, 9.70.

#### **2-amino-4-(2H-1,3-benzodioxol-5-yl)-6-phenylpyridine-3-carbonitrile (7)**

Purified by column chromatography (*n*-hexane-ethyl acetate 2:1) and then recrystallized from EtOH to give 0.236 g, 75% yield (96% purity by HPLC). MP 220–221 °C. ^1^H NMR (300 MHz, CDCl3), δ (ppm) 8.12–7.86 (m, 2H), 7.56–7.38 (m, 3H), 7.20–7.08 (m, 3H), 6.95 (d, J = 8.0 Hz, 1H), 6.06 (s, 2H), 5.33 (s, 2H). MS (EI) *m/z* (%): 315.11 (M^+^, 100), 157.52 (5). Analysis calculated for C_19_H_13_N_3_O_2_: C, 72.37; H, 4.16; N, 13.33; O, 10.15. Found: C, 72.45; H, 4.06; N, 13.49; O, 10.00.

#### **2-amino-4-cyclohexyl-6-phenylpyridine-3-carbonitrile (8)**

Purified by column chromatography (*n*-hexane-ethyl acetate 2:1) and then recrystallized from EtOH to give 0.216 g, 78% yield (98% purity by HPLC). MP 125–126 °C. ^1^H NMR (300 MHz, CDCl_3_) δ (ppm): 7.95–7.92 (m, 1H), 7.53–7.43 (m, 3H), 7.05 (s, 1H), 6.73 (s, 1H), 5.22 (s, 2H), 2.90–2.85 (m, 2H), 1.90–1.78 (m, 4H), 1.52–1.39 (m, 4H), 1.33–1.25 (m, 1H). MS (EI) *m/z* (%): 277.25 (M^+^, 74), 246.15 (56), 222.15 (100). Analysis calculated for C_18_H_19_N_3_: C, 77.95; H, 6.90; N, 15.15. Found: C, 78.03; H, 6.96; N, 15.01.

#### **2-amino-4-cyclohexyl-6-(2-fluorophenyl)pyridine-3-carbonitrile (9)**

Purified by column chromatography (*n*-hexane-ethyl acetate 2:1) and then recrystallized from EtOH to give 0.186 g, 63% yield (95% purity by HPLC). MP 126–127 °C. ^1^H NMR (300 MHz, CDCl3), δ (ppm) 7.89 (td, J = 7.8, 1.9 Hz, 1H), 7.47–7.31 (m, 1H), 7.25–7.03 (m, 3H), 5.18 (s, 2H), 2.98–2.67 (m, 1H), 1.99–1.73 (m, 5H), 1.53–1.16 (m, 5H). MS (EI) *m/z* (%): 295.15 (M^+^, 98.05), 263.05 (23.28), 251.00 (12), 240.00 (100). Analysis calculated for C_18_H_18_FN_3_: C, 73.20; H, 6.14; F, 6.43; N, 14.23. Found: C, 73.22; H, 6.17; F, 6.44; N, 14.25.

#### **2-amino-4-cyclohexyl-6-(2-methylphenyl)pyridine-3-carbonitrile (10)**

Purified by column chromatography (*n*-hexane-ethyl acetate 2:1) and then recrystallized from EtOH to give 0.236 g, 81% yield (97% purity by HPLC). MP 120–121 °C. ^1^H NMR (300 MHz, CDCl3), δ (ppm) 7.73–7.10 (m, 4H), 6.71 (s, 1H), 5.20 (s, 2H), 2.95–2.77 (m, 1H), 2.35 (s, 3H), 2.01–1.69 (m, 5H), 1.56–1.34 (m, 4H), 1.34–1.18 (m, 1H). MS (EI) *m/z* (%): 291.14 (M^+^, 100), 236.12 (48), 208.10 (91.7). Analysis calculated for C_19_H_21_N_3_: C, 78.32; H, 7.26; N, 14.42. Found: C, 78.48; H, 7.18; N, 14.34.

#### **2-amino-4-cyclohexyl-6-(thiophen-2-yl)pyridine-3-carbonitrile (11)**

Purified by column chromatography (*n*-hexane-ethyl acetate 2:1) and then recrystallized from EtOH to give 0.167 g, 59% yield (98% purity by HPLC). MP 160–162 °C. ^1^H NMR (300 MHz, CDCl3), δ(ppm) 7.63–7.62(m, 1H), 7.44 (d, J = 4.5 Hz, 1H), 7.12–7.09 (m, 1H), 6.96 (s, 1H), 5.14 (s, 2H), 2.82–2.79 (m, 1H), 1.90–1.78 (m, 5H), 1.55–1.43 (m, 4H), 1.30–1.19 (m, 1H). MS (EI) *m/z* (%): 283.04 (M^+^, 100), 251.99 (19), 228.02 (92). Analysis calculated for C_16_H_17_N_3_S: C, 67.81; H, 6.05; N, 14.83; S, 11.31. Found: C, 67.89; H, 6.13; N, 14.77; S, 11.21.

#### **2-amino-4-cyclohexyl-6-(thiophen-3-yl)pyridine-3-carbonitrile (12)**

Purified by column chromatography (*n*-hexane-ethyl acetate 2:1) and then recrystallized from EtOH to give 0.147 g, 52% yield (96% purity by HPLC). MP 145–146 °C. ^1^H NMR (300 MHz, CDCl3), δ(ppm) 7.94 (dd, J = 3.0, 1.3 Hz, 1H), 7.59 (dd, J = 5.1, 1.3 Hz, 1H), 7.38 (dd, J = 5.1, 3.0 Hz, 1H), 6.93 (s, 1H), 5.14 (s, 2H), 2.95–2.73 (m, 1H), 2.06–1.73 (m, 5H), 1.56–1.37 (m, 4H), 1.38–1.19 (m, 1H). MS (EI) *m/z* (%):(%): 283.07 (M^+^, 100), 228.04 (93), 214.96 (52).Analysis calculated for C_16_H_17_N_3_S: C, 67.81; H, 6.05; N, 14.83; S, 11.31. Found: C, 67.91; H, 6.09; N, 14.67; S, 11.33.

#### **2-amino-4-cyclohexyl-6-(furan-2-yl)pyridine-3-carbonitrile (13)**

Purified by column chromatography (*n*-hexane-ethyl acetate 2:1) and then recrystallized from EtOH to give 0.174 g, 65% yield (98% purity by HPLC). MP 177–178 °C. ^1^H NMR (300 MHz, CDCl3), δ(ppm) 7.55 (dd, J = 1.7, 0.8 Hz, 1H), 7.06 (dd, J = 3.4, 0.8 Hz, 1H), 7.03 (s, 1H), 6.54 (dd, J = 3.5, 1.8 Hz, 1H), 5.15 (s, 2H), 3.01–2.68 (m, 1H), 2.04–1.74 (m, 5H), 1.55–1.39 (m, 4H), 1.34–1.20 (m, 1H). MS (EI) *m/z* (%): 267.11 (M^+^, 100), 212.02 (69). Analysis calculated for C_16_H_17_N_3_O: C, 71.89; H, 6.41; N, 15.72; O, 5.98. Found: C, 71.91; H, 6.43; N, 15.71.

#### **2-amino-6-(2-fluorophenyl)-4-(4-methoxyphenyl)pyridine-3-carbonitrile (14)**

Purified by column chromatography (*n*-hexane-ethyl acetate 2:1) and then recrystallized from EtOH to give 0.188 g, 59% yield (97% purity by HPLC). MP 180–181 °C. ^1^H NMR (300 MHz, CDCl3), δ (ppm) 7.96 (td, J1 = 7.8, J2 = 1.9 Hz, 1H), 7.65–7.58 (m, 2H), 7.47–7.37 (m, 1H), 7.31–7.23 (m, 2H), 7.23–7.09 (m, 1H), 7.09–6.98 (m, 2H), 5.32 (s, 2H), 3.88 (s, 3H). MS (EI) *m/z* (%): 319.12 (M^+^, 100), 304.18 (12), 249.13 (16). Analysis calculated for C_19_H_14_FN_3_O: C, 71.46; H, 4.42; F, 5.95; N, 13.16; O, 5.01. Found: C, 71.48; H, 4.44; F, 5.97; O, 5.05.

#### **2-amino-4-(4-methoxyphenyl)-6-(2-methylphenyl)pyridine-3-carbonitrile (15)**

Purified by column chromatography (*n*-hexane-ethyl acetate 2:1) and then recrystallized from EtOH to give 0.205 g, 65% yield (95% purity by HPLC). MP 151–152 °C. ^1^H NMR (300 MHz, CDCl3), δ (ppm) 7.61 (d, J = 8.3 Hz, 2H), 7.40 (d, J = 7.3 Hz, 1H), 7.37–7.27 (m, 3H), 7.03 (d, J = 8.2 Hz, 2H), 6.86 (s, 1H), 5.32 (s, 2H), 3.87 (s, 3H), 2.42 (s, 3H). MS (EI) *m/z* (%): 314.10 (M^+^, 100), 271.06 (7), 208.11 (52). Analysis calculated for C_20_H_17_N_3_O: C, 76.17; H, 5.43; N, 13.32; O, 5.07. Found: C, 76.31; H, 5.33; N, 13.52; O, 4.84.

#### **2-amino-6-(furan-2-yl)-4-(4-methoxyphenyl)pyridine-3-carbonitrile (16)**

Purified by column chromatography (*n*-hexane-ethyl acetate 2:1) and then recrystallized from EtOH to give 0.198 g, 68% yield (99% purity by HPLC). MP 205–207 °C. ^1^H NMR (300 MHz, CDCl_3_) δ (ppm): 7.65–7.54 (m, 3H), 7.16 (s, 1H), 7.11 (d, *J* = 3.5 Hz, 1H), 7.03 (d, *J* = 8.8 Hz, 2H), 6.62–6.51 (m, 1H), 5.30 (s, 2H), 3.88 (s, 3H). MS (EI) *m/z* (%): 291.12 (M^+^, 100), 145.63 (5). Analysis calculated for C_17_H_13_N_3_O_2_: C, 70.09; H, 4.50; N, 14.42; O, 10.98. Found: C, 70.21; H, 4.38; N, 14.68, O, 10.73.

#### **2-amino-6-(4-hydroxyphenyl)-4-(4-methoxyphenyl)pyridine-3-carbonitrile (17)**

Purified by column chromatography (*n*-hexane- ethyl acetate 2:1) and then recrystallized from EtOH to give 0.222 g, 70% yield (99% purity by HPLC). MP 248–250 °C. ^1^H NMR (300 MHz, CDCl3), δ (ppm) 9.89 (s, 1H), 7.98 (d, J = 8.7 Hz, 2H), 7.61 (d, J = 8.7 Hz, 2H), 7.11–7.06 (m, 3H), 6.84–6.81 (m, 4H), 3.82 (s, 3H). MS (EI) *m/z* (%): 317.17 (M^+^, 100), 302.04 (6), 158.50 (14). Analysis calculated for C_19_H_15_N_3_O_2_: C, 71.91; H, 4.76; N, 13.24; O, 10.08. Found: C, 71.94; H, 4.79; N, 13.25; O, 10.11.

#### **2-amino-4,6-bis(2-fluorophenyl)pyridine-3-carbonitrile (18)**

Purified by column chromatography (*n*-hexane-ethyl acetate 2:1) and then recrystallized from EtOH to give 0.219 g, 73% yield (98% purity by HPLC). MP 180–181 °C. ^1^H NMR (300 MHz, CDCl3), δ (ppm) 8.05–7.90 (m, 1H), 7.56–7.41 (m, 2H), 7.33–7.06 (m, 6H), 5.34 (s, 2H). MS (EI) *m/z* (%): 307.06 (M^+^, 100), 279.99 (8). Analysis calculated for C_18_H_11_F_2_N_3_: C, 70.35; H, 3.61; F, 12.36, N, 13.67. Found: C, 70.37; H, 3.63; F, 12.33; N, 13.66.

#### **2-amino-6-(2-fluorophenyl)-4-(2-methoxyphenyl)pyridine-3-carbonitrile (19)**

Purified by column chromatography (*n*-hexane-ethyl acetate 2:1) and then recrystallized from EtOH to give 0.245 g, 78% yield (97% purity by HPLC). MP 187–188 °C. ^1^H NMR (300 MHz, CDCl3), δ (ppm) 7.97 (td, *J* = 7.8, 1.9 Hz, 1H), 7.52–7.35 (m, 2H), 7.31 (td, *J* = 7.2, 1.5 Hz, 1H), 7.26–7.19 (m, 2H), 7.17–6.95 (m, 3H), 5.27 (s, 2H), 3.88 (s, 3H). MS (EI) *m/z* (%): 319.12 (M^+^, 100), 290.14 (7), 138.01 (14). Analysis calculated for C_19_H_14_N_3_FO: C, 71.46; H, 4.42; F, 5.95; N, 13.16; O, 5.01. Found: C, 71.44; H, 4.43; F, 5.92; O, 5.04.

#### **2-amino-4-(2-methoxyphenyl)-6-(2-methylphenyl)pyridine-3-carbonitrile (20)**

Purified by column chromatography (*n*-hexane-ethyl acetate 2:1) and then recrystallized from EtOH to give 0.186 g, 64% yield (98% purity by HPLC). MP 181–183 °C. ^1^H NMR (300 MHz, CDCl_3_) δ (ppm): 7.47–7.40 (m, 2H), 7.32–7.28 (m, 4H), 7.09–7.02 (m, 2H), 6.86 (s, 1H), 5.29 (s, 2H), 3.88 (s, 3H), 2.43 (s, 3H). MS (EI) *m/z* (%): 315.13 (M^+^, 100), 298.16 (12), 284.09 (18), 208.10 (81.6). Analysis calculated for C_20_H_17_N_3_O: C, 76.17; H, 5.43; N, 13.32; O, 5.07. Found: C, 76.19; H, 5.41; N, 13.36; O, 5.03.

#### **2-amino-6-(furan-2-yl)-4-(2-methoxyphenyl)pyridine-3-carbonitrile (21)**

Purified by column chromatography (*n*-hexane-ethyl acetate 2:1) and then recrystallized from EtOH to give 0.244 g, 77% yield (96% purity by HPLC). MP 187–188 °C. ^1^H NMR (300 MHz, CDCl_3_), δ (ppm): 7.55 (s, 1H), 7.44 (t, *J* = 8.1 Hz, 1H), 7.30 (dd, *J* = 7.4, 1.7 Hz, 1H), 7.15–6.98 (m, 4H), 6.54 (dd, *J* = 3.3, 1.7 Hz, 1H), 5.24 (s, 2H), 3.87 (s, 3H). MS (EI) *m/z* (%): 291.10 (M^+^, 100), 262.14 (10). Analysis calculated for C_17_H_13_N_3_O_2_: C, 70.09; H, 4.50; N, 14.42; O, 10.98. Found: C, 70.11; H, 4.51; N, 14.41; O, 11.01.

#### **2-amino-6-(4-hydroxyphenyl)-4-(2-methoxyphenyl)pyridine-3-carbonitrile (22)**

Purified by column chromatography (*n*-hexane-ethyl acetate 2:1) and then recrystallized from EtOH to give 0.193 g, 60% yield (96% purity by HPLC). MP 210–212 °C. ^1^H NMR (300 MHz, DMSO-*d*_*6*_), δ (ppm): 9.91 (s, 1H), 7.93 (d, *J* = 9.0 Hz, 2H), 7.45 (t, *J* = 7.8 Hz, 1H), 7.29 (dd, *J* = 7.4, 1.7 Hz, 1H), 7.16 (d, *J* = 8.3 Hz, 1H), 7.07 (d, *J* = 7.5 Hz, 1H), 7.03 (s, 1H), 6.82 (d, *J* = 8.9 Hz, 2H), 6.77 (s, 2H), 3.77 (s, 3H). MS (EI) *m/z* (%): 317.13 (M^+^, 100), 300.09 (8), 286.11 (6).Analysis calculated for C_19_H_15_N_3_O_2_: C, 71.91; H, 4.76; Cl, 13.24; O, 10.08. Found: C, 71.92; H, 4.74; Cl, 13.27; O, 10.05.

#### **2-amino-4-(2-chlorophenyl)-6-(4-hydroxyphenyl)pyridine-3-carbonitrile (23)**

Purified by column chromatography (*n*-hexane-ethyl acetate 2:1) and then recrystallized from EtOH to give 0.179 g, 59% yield (98% purity by HPLC). MP 215–217 °C. ^1^H NMR (300 MHz, DMSO-*d*_*6*_), δ (ppm): 9.90 (s, 1H), 8.16–7.22 (m, 2H), 7.69–7.30 (m, 4H), 7.16–6.50 (m, 5H). MS (EI) *m/z* (%): 320.99 (M^+^, 100), 286.04 (5). Analysis calculated for C_18_H_12_ClN_3_O: C, 67.19; H, 3.76; Cl, 11.02; N, 13.06; O, 4.97. Found: C, 67.37; H, 3.94; Cl, 11.18; N, 12.88; O, 4.63.

#### **2-amino-4,6-bis(4-hydroxyphenyl)pyridine-3-carbo-nitrile (24)**

Purified by column chromatography (*n*-hexane–ethyl acetate 2:1) and then recrystallized from EtOH to give 0.151 g, 53% yield (97% purity by HPLC). MP 299–300 °C. ^1^H NMR (300 MHz, DMSO-*d*_*6*_), δ (ppm) 9.92 (s, 2H), 8.19–7.79 (m, 2H), 7.68–7.37 (m, 2H), 7.42–6.99 (m, 1H), 7.01–6.62 (m, 6H). MS (EI) *m/z* (%): 303.06 (M^+^, 100), 184.01 (6). Analysis calculated for C_18_H_13_N_3_O_2_: C, 71.28; H, 4.32; N, 13.85; O, 10.55. Found: C, 71.40; H, 4.54; N, 13.75; O, 10.31.

#### **2-amino-4-(furan-2-yl)-6-(thiophen-3-yl)pyridine-3-carbonitrile (25)**

Purified by column chromatography (*n*-hexane-ethyl acetate 2:1) and then recrystallized from EtOH to give 0.123 g, 46% yield (95% purity by HPLC). MP 156–157 °C. ^1^H NMR (300 MHz, CDCl_3_) δ(ppm): 8.01 (dd, J = 3.0, 1.2 Hz, 1H), 7.66 (dd, *J* = 5.1, 1.2 Hz, 1H), 7.62 (dd, *J* = 1.8, 0.6 Hz, 1H), 7.48 (dd, *J* = 3.6, 0.6 Hz, 1H), 7.45 (s, 1H), 7.40 (dd, *J* = 5.1, 3.0 Hz, 1H), 7.40 (dd, *J* = 5.1, 3.0 Hz, 1H), 6.61 (dd, *J* = 3.6, 1.8 Hz, 1H), 5.26 (s, 2H). MS (EI) *m/z* (%): 267.06 (M^+^, 100), 237.98 (6), 210.99 (7). Analysis calculated for C_14_H_9_N_3_OS: C, 62.91; H, 3.39; N, 15.72; O, 5.99; S, 11.99. Found: C, 63.11; H, 3.47; N, 15.58; O, 5.97; S, 11.87.

### Pharmacological evaluation of novel 4,6-substituted 2-amino-pyridin-3-carbonitriles

Pharmacological evaluation was performed in a radioligand binding competition assay, using A_1_, A_2A_, A_2B_, and A_3_ human receptors expressed in transfected CHO (A_1_), HeLa (A_2A_ and A_3_), and HEK-293 (A_2B_) according to the procedure reported by Bosch et al. [78].

 The activity measurements against the phosphodiesterases PDE7A, PDE7B, PDE9A and PDE10A were performed using AD293 cells that were transiently and separately transfected with human PDE7A, PDE7B, PDE9A, and PDE10A following the procedure described by Shipe et al. [43]. The IC_50_ values were obtained by fitting the data with non-linear regression using Prism 2.1 software (GraphPad, San Diego, CA) [79], and the reported results are the mean of 3 experiments (n = 3) each performed in duplicate.

## Additional files

**Additional file 1.** Supplementary data describing substructural analysis of extracted ChEMBL compounds, statistical analysis of enriched target prediction of RECAP compounds, separation in medians of active/inactive docking score distributions for the docking models, computed logP and tPSA values and selectivity profiling data for compounds **1–25**, docking parameters used, scripts for compound extraction from the ChEMBL database, computation of Mann–Whitney test and F_1_ scores.

**Additional file 2.** Coordinates of the A_1_R homology model.

**Additional file 3.** CSV file of computed F_1_ scores of the A_1_R docking model.

**Additional file 4.** CSV file of computed F_1_ scores of the A_2A_R docking model.

**Additional file 5.** CSV file of computed F_1_ scores of the PDE10A docking model.

## Abbreviations

AR: adenosine receptor
A_1_R: A_1_ adenosine receptor
A_2A_R: A_2A_ adenosine receptor
A_2B_R: A_2B_ adenosine receptor
A_3_R: A_3_ adenosine receptor
PDE10A: cAMP and cAMP-inhibited cGMP 3′,5′-cyclic phosphodiesterase 10A
cAMP: cyclic adenosine monophosphate
PDE7A: high affinity cAMP-specific 3′,5′-cyclic phosphodiesterase 7A
PDE7B: cAMP-specific 3′,5′-cyclic phosphodiesterase 7B
PDE9A: high affinity cGMP-specific 3′,5′-cyclic phosphodiesterase 9A
PDGFR: platelet-derived growth factor receptor
VEGFR: vascular endothelial growth factor receptor
ABL1: tyrosine-protein kinase ABL1
SRC: proto-oncogene tyrosine-protein kinase Src
EGFR: epidermal growth factor receptor
HER2: receptor tyrosine-protein kinase erbB-2
MAO-A: amine oxidase [flavin-containing] A
MAO-B: amine oxidase [flavin-containing] B
AChE: acetylcholinesterase
BuChE: butyrylcholinesterase
H3-R: histamine H3 receptor
HMT: histone methyltransferases
hCB2R: human cannabinoid receptor 2
EtOH: ethanol
