# Supplementary material for: Computer-aided design of multi-target ligands at A1R, A2AR and PDE10A, key proteins in neurodegenerative diseases
Source: J Cheminform. 2017 Dec 30;9:67. doi: 10.1186/s13321-017-0249-4 (PMC5748027; doi:10.1186/s13321-017-0249-4)
Supplement: Supplementary file 1 — Additional file 1. Supplementary data describing substructural analysis of extracted ChEMBL compounds, statistical analysis of enriched target prediction of RECAP compounds, separation in medians of active/inactive docking score distributions for the docking models, computed logP and tPSA values and selectivity profiling data for compounds 1–25, docking parameters used, scripts for compound extraction from the ChEMBL database, computation of Mann–Whitney test and F1 scores. [file 13321_2017_249_MOESM1_ESM.docx]

**Supporting Information**

**Computer-Aided Design of Multi-target Ligands at A_1_R, A_2A_R and PDE10A, Key Proteins in Neurodegenerative Diseases**

*Leen Kalash,^1,§^ Cristina Val,^2,3,§^ Jhonny Azuaje,^2^ María I. Loza,^3^ Fredrik Svensson,^1,4^* *Azedine Zoufir,^1^ Lewis Mervin,^1,5^ Graham Ladds,^6^José Brea,^3^ Robert Glen,^1,7^ Eddy Sotelo,^2,*^ Andreas Bender^1,*^*

1. Centre for Molecular Informatics, Department of Chemistry, University of Cambridge, Lensfield Road, Cambridge,CB21EW, United Kingdom

2. Center for Research in Biological Chemistry and Molecular Materials (CIQUS), University of Santiago de Compostela, Santiago de Compostela, 15782, Spain

3. Center for Research in Molecular Medicine and Chronic Diseases (CIMUS), University of Santiago de Compostela, Santiago de Compostela, 15782, Spain

4. IOTA Pharmaceuticals Ltd, St Johns Innovation Centre, Cowley Road, Cambridge CB4 0WS, United Kingdom***.***

5. Discovery Sciences, AstraZeneca R&D, Cambridge Science Park, United Kingdom

6. Department of Pharmacology, University of Cambridge, Tennis Court Road, Cambridge, CB21QJ, United Kingdom

7. Department of Surgery and Cancer - Division of Computational and Systems Medicine, Imperial College London, London, United Kingdom

Multi-target ligands, Adenosine receptor ligands, PDE10A inhibitors, Target prediction, Drug Design, Docking, QSAR

**Table of Contents**

I. Figures..............................................................................................................................................**S2-S6**

Figure S1. Substructural analysis of extracted ChEMBL compounds……..…………………...**S2**

Figure S2. Statistical Analysis of enriched target prediction of RECAP combinations………..**S3**

Figure S3. Docking studies of the overlaid compounds **16** and **21** with PDE10A……………..**S4**

Figure S4. CNS filtering of compounds **8** and **16**……………………………………………....**S5**

Figure S5. Separation in medians of active/inactive docking score distributions

for the docking models…………….............................................................................................**S6**

II. Tables …………………………………………...................................................................... ….**S7-S17**

Table S1. Computed LogP and tPSA values for Compounds **1**-**25**…………………………….**S7**

Table S2. Selectivity profiling data for Compounds **1-25**…………………………………….**S12**

Table S3. Docking parameters used…………………………………………………………...**S17**

III. Scripts................................................................................................................................... ….**S18-S22**

Script1. SQL script for extracting ChEMBL compounds……………………………………..**S18**

Script2. R script for the computation of Mann-Whitney test………………………………….**S19**

Script3. Python script for the computation of F_1_ scores….……. ……………………………..**S20**

IV. References………………………………………………………………………………….……….**S23**

***I. Figures***

*
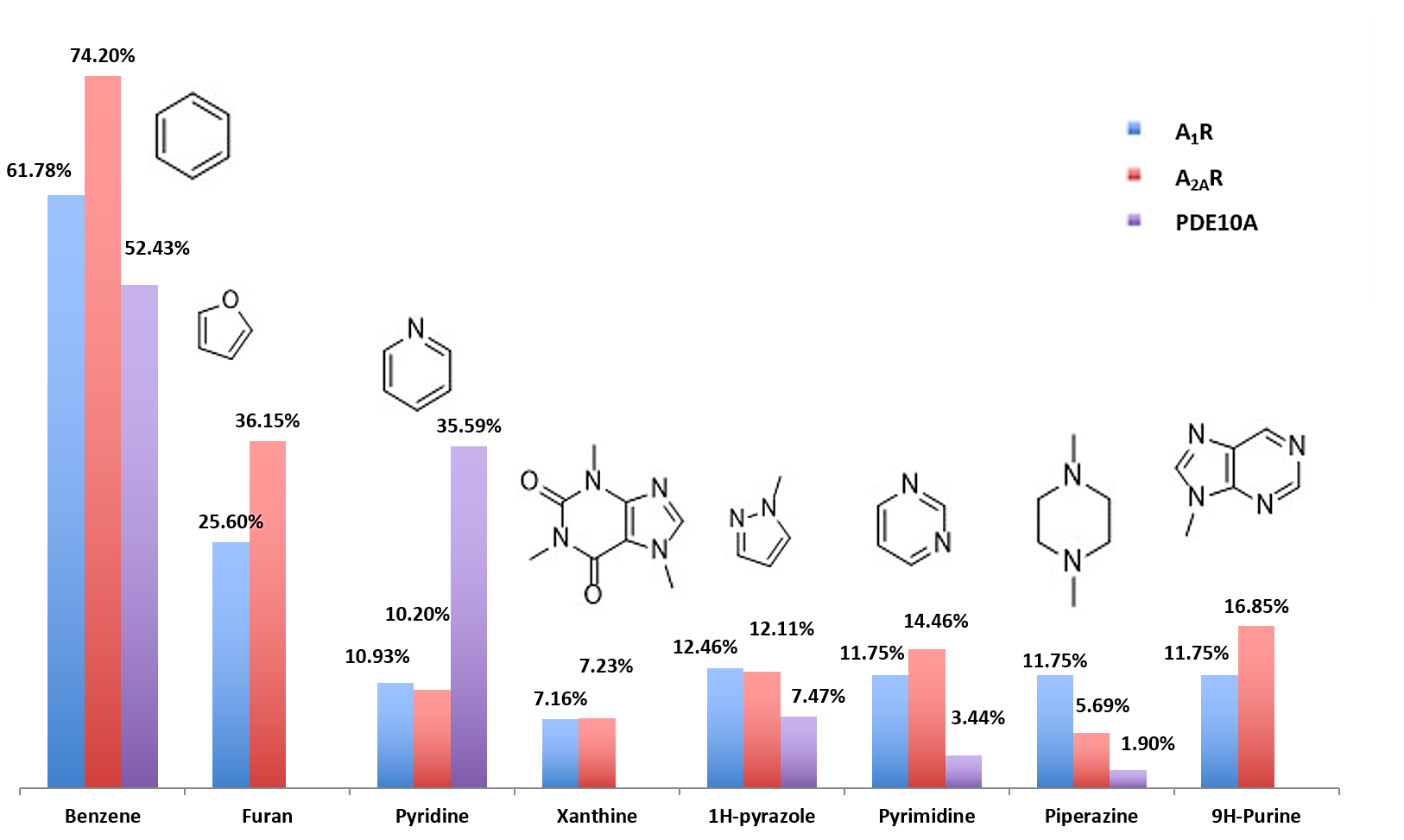
*

**Figure S1**. Percentage occurrence of the most frequent substructures in the A_1_R, A_2A_R, and PDE10A ChEMBL compounds (K_i_ and IC_50_ ≤ 1 μ M) reveals the following substructures for A_1_R, A_2A_R, and PDE10A inhibitors: benzene, furan, pyridine, xanthine, 1H-pyrazole, pyrimidine, piperazine, and 9H-purine, in cases where no percentage is displayed for a particular target, this means that the substructure does not appear among the top 30 for the set of compounds involved

**
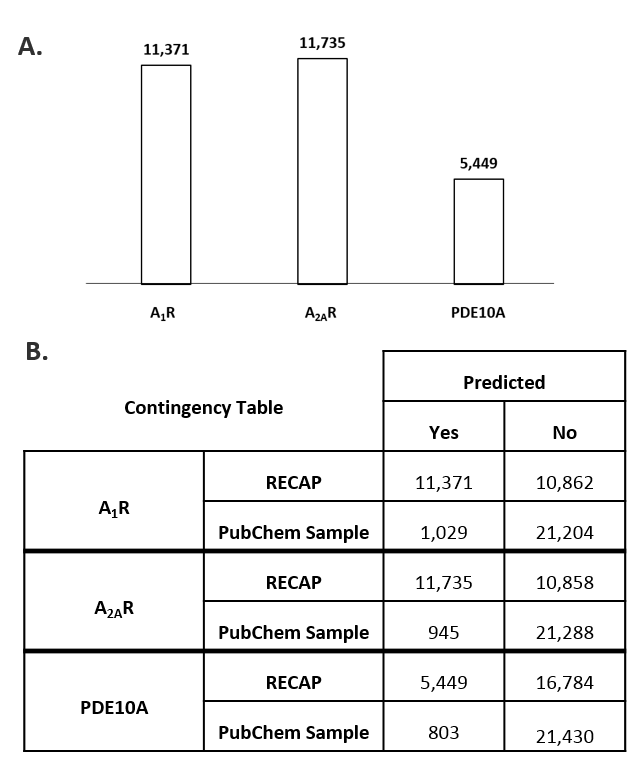
**

**Figure S2***.* A. A_1_R, A_2A_R and PDE10A were predicted as enriched targets with an estimation score equal to 0 (enriched) and average ratios less than 0.1 (enriched) for the focused RECAP library, where the percentage of RECAP compounds that were predicted as actives against the A_1_R, A_2A_R and PDE10A targets are: 51.1%, 52.8%, and 24.5%, with Chi-squared p values ≥ 0.0001 and Chi-squared statistic of 11958.8, 12842.1, and 4015.7, respectively B**.** The results of the contingency table for the Chi-squared calculation are passed to Scipy,^1^ for example the calculation for A_1_R is scipy.stats.chi2_contingency ([[1029, 21204], [11371, 10862]]).


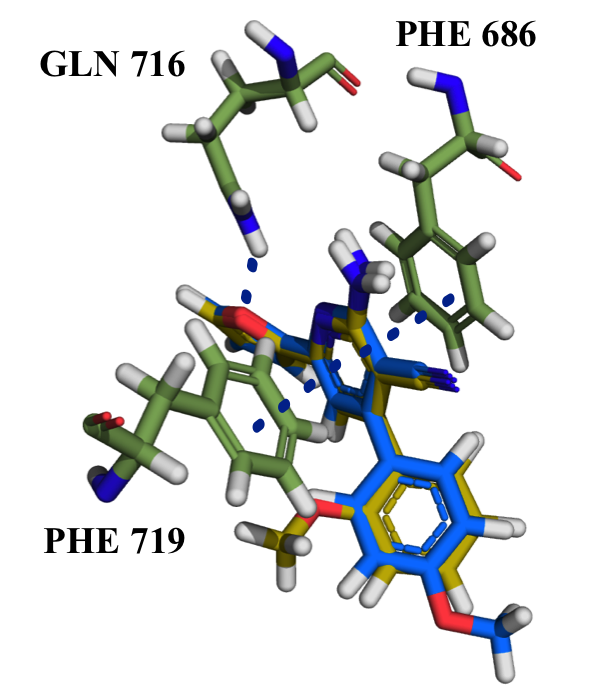


**Figure S3**. Docking studies predicted molecular interactions for the overlaid compounds **16** and **21** with PDE10A: π-stacking of the pyridine rings with Phe_686_ and Phe_719_, and H-bonding with Gln_716_ *via* their overlaid furan rings at position 6 of the pyridine ring


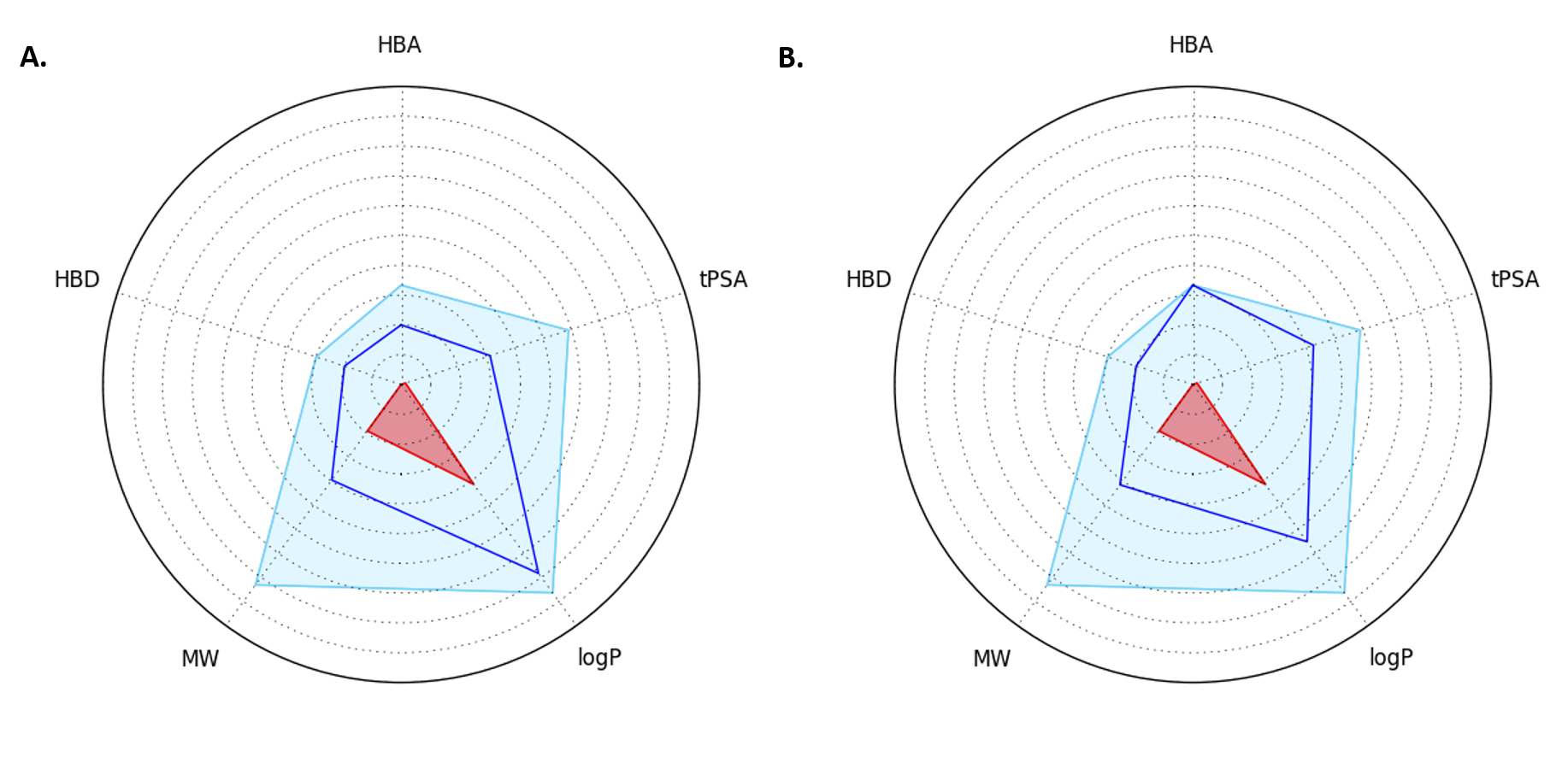


**Figure S4.** Compounds **8** and **16** (represented in 3A and 3B respectively), have passed the CNS filter which takes into consideration the assessment of their ability to pass the blood brain barrier, values of compounds **8** and **16** (blue line) fall within the CNS filter area (light blue)

***
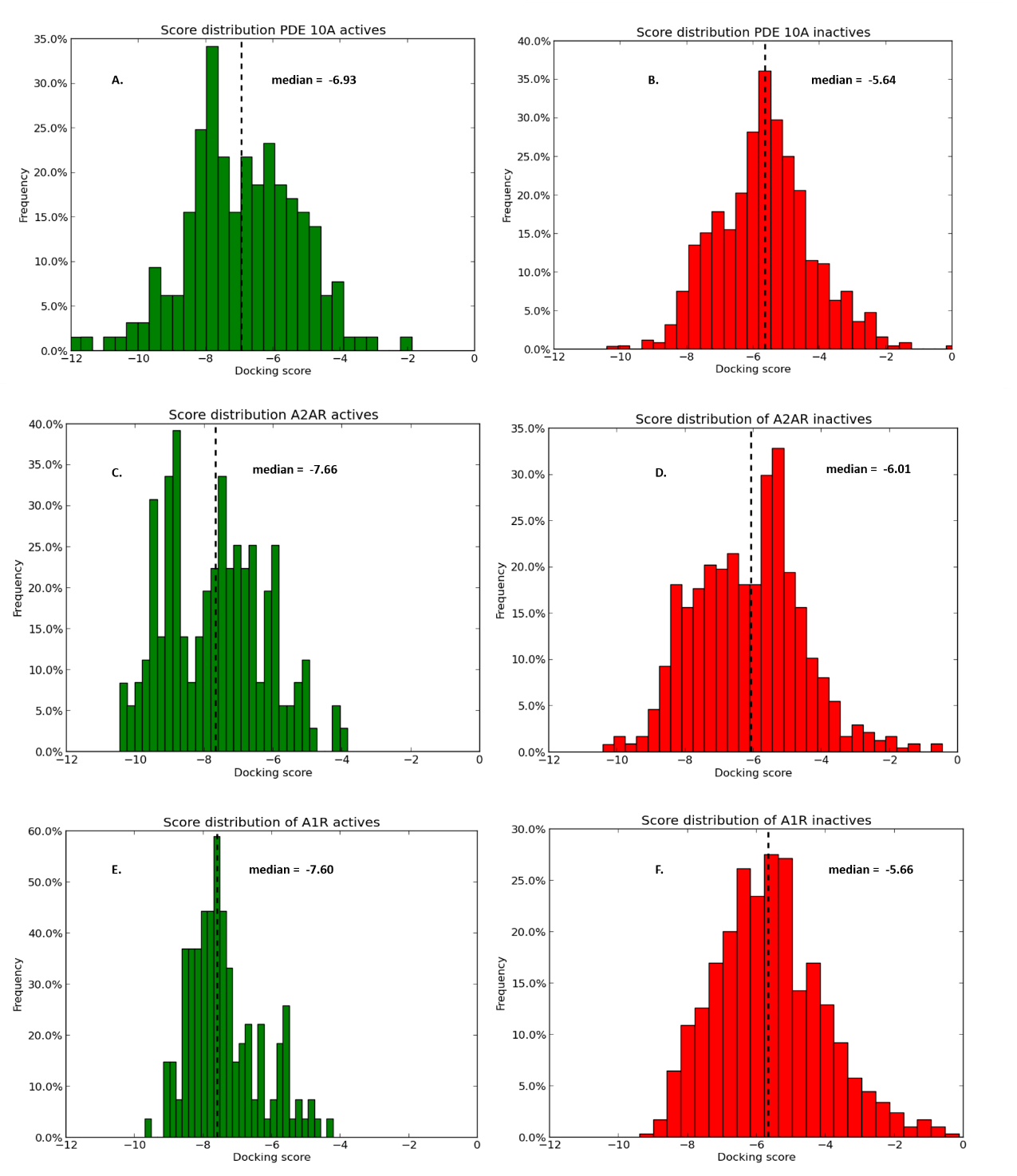
***

**Figure S5**. The separation in the medians obtained for the three docking models A. -6.93 (ChEMBL actives) B. -5.64 (PubChem in-actives) for the PDE10A docking model, C. -7.66 (ChEMBL actives) D. -6.01 (PubChem inactives) for the A_2A_R docking model, E. -7.60 (ChEMBL actives) F. -5.66 (PubChem inactives) for the A_1_R docking model, indicating that actives are enriched in the three docking models

***II. Tables***

**Table S1.** Physicochemical properties (LogP and tPSA) computed for the synthesized 4,6-substituted 2-amino-pyridin-3-carbonitriles using FAFDrug3 ADME-Tox^2^

| **Compound** | **R_4_** | **R_6_** | **logP** | **tPSA (Å^2^)** |
| --- | --- | --- | --- | --- |
| **1** | **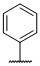** | **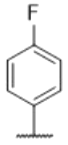** | 4.2 | 62.7 |
| **2** |  | **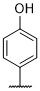** | 3.7 | 82.9 |
| **3** |  | **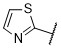** | 3.1 | 103.8 |
| **4** |  | **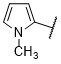** | 2.8 | 67.6 |
| **5** | **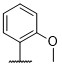** | **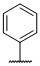** | 4.0 | 71.9 |
| **6** | **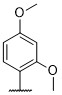** |  | 4.0 | 81.2 |
| **7** | **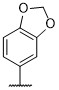** |  | 3.9 | 81.2 |
| **8** | **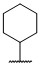** | **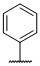** | 5.0 | 62.7 |
| **9** |  | **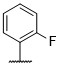** | 5.1 | 62.7 |
| **10** |  | **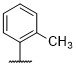** | 5.3 | 62.7 |
| **11** |  | **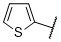** | 4.7 | 90.9 |
| **12** |  | **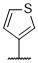** | 4.7 | 90.9 |
| **13** |  | **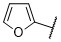** | 4.1 | 75.8 |
| **14** | **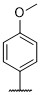** | **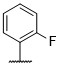** | 4.1 | 71.9 |
| **15** |  | **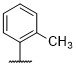** | 4.4 | 71.9 |
| **16** |  | **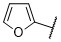** | 3.1 | 85.1 |
| **17** |  | **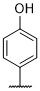** | 3.6 | 92.2 |
| **18** | **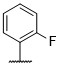** | **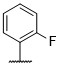** | 4.3 | 62.7 |
| **19** | **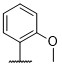** | **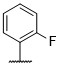** | 4.3 | 62.7 |
| **20** |  | **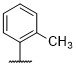** | 4.4 | 71.9 |
| **21** |  | **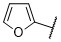** | 3.1 | 85.1 |
| **22** |  | **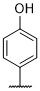** | 3.7 | 92.2 |
| **23** | **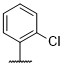** | **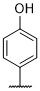** | 4.3 | 82.9 |
| **24** | **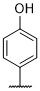** |  | 3.4 | 103.2 |
| **25** | **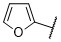** | **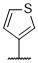** | 2.8 | 104.1 |

**Table S2**. Percentage inhibition of the synthesized 4,6-substituted 2-amino-pyridin-3-carbonitriles at 10µM (PDE7A, PDE7B, PDE9A), or IC_50_ (µM) and percentage displacement at 0.1µM (A_2B_R and A_3_R), or K_i_

| **Compound** | **R_4_** | **R_6_** | **A_2B_R** | **A_3_R** | **PDE7A** | **PDE7B** | **PDE9A** |
| --- | --- | --- | --- | --- | --- | --- | --- |
| **1** | **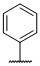** | **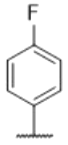** | 2% | 27% | 9% | 28% | 1% |
| **2** |  | **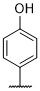** | 2% | 2% | 47% | 39% | 1% |
| **3** |  | **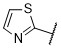** | 3% | 6% | 24% | 36% | 5% |
| **4** |  | **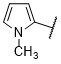** | 5% | 13% | 50% | 48% | 2% |
| **5** | **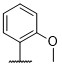** | **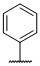** | 5% | 26% | 24% | 30% | 3% |
| **6** | **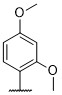** |  | 1% | 6% | 51% | 27% | 1% |
| **7** | **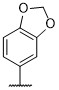** |  | 1% | 2% | 22% | 8% | 19% |
| **8** | **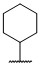** | **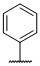** | 1% | 13% | 26% | 55% | 30% |
| **9** |  | **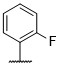** | 2% | 7% | 19% | 34% | 1% |
| **10** |  | **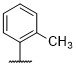** | 1% | 28% | 31% | 35% | 9% |
| **11** |  | **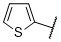** | 1% | 26% | 33% | 45% | 6% |
| **12** |  | **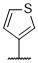** | 2% | 14% | 29% | 60% | 3% |
| **13** |  | **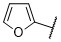** | 3% | 19% | 44% | 41% | 4% |
| **14** | **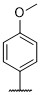** | **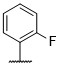** | 1% | 27% | 22% | 20% | 7% |
| **15** |  | **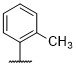** | 4% | 17% | 57% | 55% | 16% |
| **16** |  | **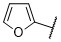** | 33% | 21% | 3.4 ± 0.4 µM | 30% | 2% |
| **17** |  | **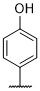** | 1% | 8% | 3.5 ± 0.4 µM | 44% | 7% |
| **18** | **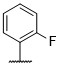** | **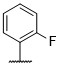** | 1% | 1% | 25% | 36% | 0% |
| **19** | **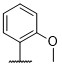** | **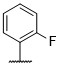** | 2% | 1% | 49% | 42% | 19% |
| **20** |  | **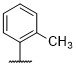** | 3% | 6% | 31% | 23% | 12% |
| **21** |  | **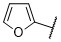** | 2% | 23% | 15.1 ± 0.6 µM | 30% | 17% |
| **22** |  | **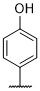** | 1% | 2% | 63% | 16% | 9% |
| **23** | **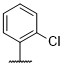** | **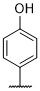** | 8% | 1% | 1.8 ± 0.3 µM | 7.3 ± 0.3 µM | 25% |
| **24** | **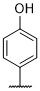** |  | 1% | 1% | 47% | 38% | 7% |
| **25** | **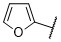** | **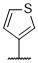** | 5% | 2% | 48% | 4.7 ± 0.4 µM | 14% |

**Table S3.** Glide^3^ docking parameters used for the A_1_R, A_2A_R, and PDE10A models were deduced from docking experiments using known actives and inactives against each protein model

| Docking model | A_1_R | A_2A_R | PDE10A |
| --- | --- | --- | --- |
| Precision option | Standard precision (SP)^a^ | Standard precision (SP)^a^ | Extra precision (XP)^a^ |
| Ligand sampling option | Flexible ^b^ | Flexible ^b^ | Flexible ^b^ |

*a* SP option allows better coverage of conformational space whereas XP option gives higher accuracy on docked poses *b* Flexible ligand sampling is a default choice, which generates conformations internally during the docking process

***III. Scripts***

**Script1.** SQL script for extracting human A_1_R (2,860), A_2A_R (3,566) agonists and antagonists and PDE10A inhibitors (843) from the ChEMBL 20 database with K_i_ and IC_50_ values ≤ 1µM

select cs.accession, td.pref_name, md.chembl_id, ac.standard_value, ac.standard_units from component_sequences cs, target_components tc, target_dictionary td, assays asa, activities ac, molecule_dictionary md where cs.component_id = tc.component_id and tc.tid = td.tid and td.tid = asa.tid and asa.assay_id = ac.assay_id and ac.molregno = md.molregno and ac.standard_type in ('IC50','Ki') and ac.standard_value ≤ 1000 and cs.accession in ('P30542 ','P29274 ','Q9Y23 ');

**Script2.** R script for the Mann-Whitney test^4^ performed on the active and inactive docking score distributions of each target, the differences in medians were significant with p values < 0.05.

actives_A_1_R = read.csv('home/user_name/A_1_R_actives_homology.csv')

inactives_A_1_R = read.csv('home/user_name/A_1_R_inactives_homology.csv')

wilcox.test(actives_ A_1_R $docking.score,inactives_ A_1_R $docking.score)

#data: actives_ A_1_R $docking.score and inactives_ A_1_R $docking.score

#W = 18594, p-value < 2.2e^-16^

actives_ A_2a_R = read.csv('home/user_name/A_2A_R_selective_antagonists.csv')

inactives_ A_2A_R= read.csv('home/user_name/A_2A_R _in_actives.csv')

wilcox.test(actives_ A_2A_R $docking.score,inactives_ A_2A_R $docking.score)

#data: actives_ A_2A_R $docking.score and inactives_ A_2A_R$docking.score

#W = 28199, p-value < 2.2e^-16^

actives_PDE10A = read.csv('home/user_name/PDE10A_actives.csv')

inactives_PDE10A = read.csv('home/user_name/PDE10A_inactives.csv')

wilcox.test(actives_PDE10A$docking.score,inactives_PDE10A$docking.score)

#data: actives_PDE10A$docking.score and inactives_PDE10A$docking.score

#W = 60873, p-value = 0.02964

**Script3.** Python script for the computation of the F_1_ score for all the docking scores of the ChEMBL actives and PubChem inactives for each of the A_1_R, A_2A_R, and PDE 10A docking models

import numpy as np

with open('/home/user_name/input_file_docking_model_actives.csv','r') as f:

header = f.next()

actives = []

for line in f:

line = line.strip()

line_list = line.split(',')

data = float(line_list[1])

actives.append(data)

with open('/home/user_name/input_file_docking_model_inactives.csv','r') as f:

header = f.next()

inactives = []

for line in f:

line = line.strip()

line_list = line.split(',')

data = float(line_list[1])

inactives.append(data)

all = actives+inactives

all= np.array(all[1:-1])

all.sort()

actives = np.array(actives)

inactives = np.array(inactives)

f1score = []

for t in all:

fp = float(len(inactives[inactives < t]))

fn = float(len(actives[actives > t]))

tp = float(len(actives[actives < t]))

tn = float(len(inactives[inactives > t]))

f1score.append(2*(tp / (tp+fn+fp)))

with open('/home/user_name/F1_score_thresholds.csv','w') as f:

f.write('Threshold, f1_score\n')

for i in xrange(len(all)):

f.write(str(all[i])+','+str(accur+str(f1score[i])'+'\n')

***IV. References***

1. Jones, E.; Oliphant, T.; Peterson, P. SciPy: Open Source Scientific Tools for Python, 2001. http://www.scipy.org/ (accessed 2017-02-27).
2. Lagorce D, Sperandio O, Baell JB, Miteva MA, Villoutreix BO (2015) FAF-Drugs3: A Web server for Compound Property Calculation and Chemical Library Design. Nucleic Acids Res 43:W200–W207.

(3) Halgren TA, Murphy RB, Friesner RA, Beard HS, Frye LL, Pollard WT, Banks JL (2004) Glide : A New Approach for Rapid , Accurate Docking and Scoring . 2 . Enrichment Factors in Database Screening. J Med Chem 47:1750–1759.

(4) R Core Team. R: A Language and Environment for Statistical Computing (Version 3.2.4). 2016.
